# Screening of viral-vectored *P*. *falciparum* pre-erythrocytic candidate vaccine antigens using chimeric rodent parasites

**DOI:** 10.1371/journal.pone.0254498

**Published:** 2021-07-12

**Authors:** Surendra Kumar Kolli, Ahmed M. Salman, Jai Ramesar, Severine Chevalley-Maurel, Hans Kroeze, Fiona G. A. Geurten, Shinya Miyazaki, Ekta Mukhopadhyay, Catherin Marin-Mogollon, Blandine Franke-Fayard, Adrian V. S. Hill, Chris J. Janse

**Affiliations:** 1 Department of Parasitology, Leiden University Medical Center, Leiden, Netherlands; 2 Nuffield Department of Medicine, The Jenner Institute, University of Oxford, Oxford, United Kingdom; INSERM, FRANCE

## Abstract

To screen for additional vaccine candidate antigens of *Plasmodium* pre-erythrocytic stages, fourteen *P*. *falciparum* proteins were selected based on expression in sporozoites or their role in establishment of hepatocyte infection. For preclinical evaluation of immunogenicity of these proteins in mice, chimeric *P*. *berghei* sporozoites were created that express the *P*. *falciparum* proteins in sporozoites as an additional copy gene under control of the *uis4* gene promoter. All fourteen chimeric parasites produced sporozoites but sporozoites of eight lines failed to establish a liver infection, indicating a negative impact of these *P*. *falciparum* proteins on sporozoite infectivity. Immunogenicity of the other six proteins (SPELD, ETRAMP10.3, SIAP2, SPATR, HT, RPL3) was analyzed by immunization of inbred BALB/c and outbred CD-1 mice with viral-vectored (ChAd63 or ChAdOx1, MVA) vaccines, followed by challenge with chimeric sporozoites. Protective immunogenicity was determined by analyzing parasite liver load and prepatent period of blood stage infection after challenge. Of the six proteins only SPELD immunized mice showed partial protection. We discuss both the low protective immunogenicity of these proteins in the chimeric rodent malaria challenge model and the negative effect on *P*. *berghei* sporozoite infectivity of several *P*. *falciparum* proteins expressed in the chimeric sporozoites.

## Introduction

The most advanced malaria vaccine is the sub-unit vaccine RTS,S that is based on the immunodominant sporozoite surface antigen, circumsporozoite protein (CSP), fused to hepatitis B virus surface antigen (HBsAg) [1]. This sub-unit vaccine was formulated with the liposomal adjuvant system AS01 from GlaxoSmithKline to target the sporozoite/liver stage of *P*. *falciparum* and has advanced to Phase IV clinical trials [1–4]. In field studies, the efficacy of RTS,S against clinical malaria has been modest; between 30% and 40% in children between the ages of 5 and 17 months and vaccine efficacy rapidly declined over time [5]. Recently a new candidate vaccine targeting CSP has been assessed for efficacy. This vaccine, R21 (also containing HBsAg fused to CSP but with adjuvant Matrix-M) showed an efficacy of around 77% in children aged 5-17^th^ months in a phase2b trial [6]. The RTS,S and R21 vaccines are aimed at inducing high titre antibodies to block the sporozoites prior to infection of hepatocytes. An alternative sub-unit vaccination strategy is the induction of high numbers of CD8+ T cells to kill infected hepatocytes. The most successful regimen to date has been the use of viral vectors, expressing the antigen in a heterologous prime-boost regimen, as for the ME-TRAP vaccine. The ME-TRAP vaccine combines the pre-erythrocytic antigen thrombospondin-related adhesion protein (TRAP) with a multi-epitope string (ME) and is delivered via the viral vectors chimpanzee adenovirus 63 (ChAd63) and modified vaccinia virus Ankara (MVA) [7–9]. Whilst this vaccine displays moderate levels of efficacy in naive-adults, it induces high CD8+ T cell responses.

While these findings with different pre-erythrocytic sub-unit vaccine approaches are encouraging, additional improvements may be required to accomplish a more protective vaccine formulation. A number of approaches are being assessed with the aim of increasing the efficacy of sub-unit vaccines, including the use of new adjuvants or different sub-unit vaccination platforms [10–14] and delivery of *P*. *falciparum* pre-erythrocytic antigens using whole sporozoites of rodent malaria parasites [15–17]. In addition, a likely way to increase the effectiveness of sub-unit malaria vaccines is the development of formulations incorporating more than one parasite antigens [18]. In addition to the CSP and TRAP, several other pre-erythrocytic antigens have been identified that show promising immunogenicity in preclinical evaluation, for example CelTOS [19–21]. For preclinical evaluation of protective immunity induced by human malaria pre-erythrocytic antigens often murine malaria models are used, including the use of transgenic rodent parasites that express the human *Plasmodium* antigens for challenge of immunized mice [22–24]. Such transgenic rodent parasites have also been used to screen for novel pre-erythrocytic antigens. For example, Longley et al. [25] tested 10 viral-vectored pre-erythrocytic *P*. *falciparum* vaccine candidates for protective efficacy by challenge of immunized mice with chimeric rodent parasites expressing the *P*. *falciparum* antigens. In the Longley study two antigens were identified, LSA1 and LSAP2, that induced significantly higher protective immune responses in inbred BALB/c as well as in outbred CD-1 mice than the two leading pre-erythrocytic *P*. *falciparum* vaccine antigens CSP and TRAP [25, 26].

In this study we have used viral-vectored vaccines expressing *P*. *falciparum* proteins in combination with the chimeric rodent malaria challenge model to screen an additional six pre-erythrocytic vaccine candidate antigens for protective immunity.

## Results

### Selection of fourteen *P*. *falciparum* pre-erythrocytic vaccine candidate antigens

In a previous study we selected ten pre-erythrocytic *P*. *falciparum* proteins for evaluation of immunogenicity, using a combination of immunization of mice with viral vectored vaccines followed by challenge with chimeric rodent malaria *P*. *berghei* parasites expressing the cognate *P*. *falciparum* antigen [25]. To screen for additional vaccine candidate antigens, we selected fourteen pre-erythrocytic proteins in this study (Table 1). The main criteria for selection of the novel proteins were the availability of evidence from published literature for sporozoite surface localization or for their involvement in hepatocyte infection. We selected proteins located at the sporozoite surface in order to induce immune responses targeting the sporozoites that are in the circulation before they invade a hepatocyte. In addition, proteins involved in hepatocyte infection were selected to induce immune responses that block hepatocyte invasion or target infected hepatocytes presenting epitopes of the selected proteins.

**Table 1:** Selected pre-erythrocytic *P*. *falciparum* proteins.

Protein name	Product name	Pf Gene ID	Pb Gene ID	SP	TM	Localisation	infectious chimeric spz^1^
HT	Hexose transporter	PF3D7_0204700	PBANKA_0302500	No	Yes	Spz surface ^[^^31^^,^ ^38^^]^	yes
SPELD	Sporozoite surface protein essential for liver stage development	PF3D7_1137800	PBANKA_0910900	No	Yes	Spz surface ^[^^27^^,^ ^34^^]^	yes
SIAP2	Sporozoite invasion-associated protein 2	PF3D7_0830300		Yes	Yes	Spz surface ^[^^33^^]^	yes
SPATR	Secreted protein with altered thrombospondin repeat domain	PF3D7_0212600	PBANKA_0309500	Yes	No	Spz surface ^[^^28^^]^	yes
RP-L3	60S ribosomal protein L3	PF3D7_1027800	PBANKA_0511900	No	No	cytoplasm spz/ liver stage ^[^^46^^]^	yes
ETRAMP 10.3	Early transcribed membrane protein 10.3	PF3D7_1016900	PBANKA_0501200	No	Yes	PV ^[^^45^^]^	yes
GEST	Gamete egress and sporozoite traversal protein	PF3D7_1449000	PBANKA_1312700	Yes	No	Spz surface ^[^^34^^,^ ^35^^]^	no
SSP3	Sporozoite surface protein 3	PF3D7_0812300	PBANKA_1425200	Yes	Yes	Spz surface ^[^^30^^,^ ^34^^,^ ^36^^]^	no
SIAP1	Sporozoite invasion-associated protein 1	PF3D7_0408600	PBANKA_1006200	Yes	No	Spz surface ^[^^29^^,^ ^34^^]^	no
MAEBL	Membrane associated erythrocyte binding-like protein	PF3D7_1147800	PBANKA_0901300	Yes	Yes	Spz surface ^[^^32^^,^ ^37^^]^	no
P36	6-cysteine protein P36	PF3D7_0404400	PBANKA_1002100	No	Yes	Micronemes, PV ^[^^43^^]^	no
P52	6-cysteine protein P52	PF3D7_0404500	PBANKA_1002200	No	Yes	Micronemes, PV	no
PLP1/ SPECT2	Perforin-like protein 1	PF3D7_0408700	PBANKA_1006300	Yes	No	Micronemes, PV ^[^^34^^,^ ^39^^]^	no
B9	6-cysteine protein B9	PF3D7_0317100	PBANKA_0808100	No	Yes	PV/PPM ^[^^40^^]^	no

^1^ Sporozoites (spz) infectivity of chimeric *P*. *berghei* parasites expressing the *P*. *falciparum* protein Pf: *P*. *falciparum*; Pb: *P*. *berghei*; SP: signal peptide; TM: transmembrane domain; Spz: sporozoites; PV: parasitophorous vacuole; PPM: parasite plasma membrane

Thirteen of the fourteen selected *P*. *falciparum* proteins have orthologues in *P*. *berghei*; only SIAP2 has been identified only in primate malaria and is absent in rodent parasites (PlasmoDB v.46, www.plasmodb.org). Thirteen proteins possess either a signal peptide (SP) or a transmembrane domain (TM) and only RPL3 lacks both SP and TM (Table 1).

For six proteins, SPELD, GEST, SSP3, SPATR, HT, MAEBL, SIAP1 and SIAP2, evidence has been presented for a sporozoite surface location [27–37]. Absence of SPELD, a sporozoite- and liver-specific protein, leads to abrogation of liver stage maturation [27]. GEST plays a role in egress of gametes from the infected RBC and in sporozoite traversal [34, 35]. SSP3, a sporozoite-and liver-stage specific protein, plays a role in sporozoite gliding motility and liver stage development [30, 36]. SPATR is expressed both in blood stages and sporozoites. Recombinant SPATR binds strongly to HepG2 cells *in vitro* and antibodies raised against this protein blocked sporozoite invasion of cultured hepatocytes [28]. HT is expressed on the surface of multiple life cycle stages, including sporozoites [31, 34, 38]. MAEBL is expressed both in blood stages and sporozoites [32, 37]. *P*. *falciparum* sporozoites lacking MAEBL showed reduced invasion of hepatocytes [37]. Antibodies raised against this protein blocked *P*. *yoelii* sporozoite invasion of cultured hepatocytes [32]. SIAP1 plays a role in sporozoite motility and sporozoites lacking SIAP1 show a defect in invading salivary glands [29]. Antibodies against both SIAP1 and SIAP2 reduced sporozoite cell traversal and infection of cultured hepatocytes [29, 33]. The sporozoite-specific protein, SPECT2, is located in the micronemes and is involved in traversal of the sinusoidal cell layer prior to hepatocyte-infection. Sporozoites that lack SPECT2 lost the ability to migrate through hepatocytes and showed reduced hepatocyte invasion [39]. Three proteins P36, P52 and B9, play a role in establishment of a parasitophorous vacuole (PV) in the infected hepatocyte [40–44]. P36 and P52 proteins, like SPECT2 are located in the micronemes of sporozoites, whereas B9 is expressed in early liver stages. Parasites lacking P36, P52 or B9 arrest liver stage development soon after invasion of hepatocytes [40, 43, 44]. ETRAMP10.3, is located in the PV membrane of both blood and liver stages [45]. The last protein, RPL3, is a component of the 60S large ribosomal subunit present in the cytoplasm [46]. It was selected based on its identification to induce cytotoxic T lymphocyte (CTL) responses mediated by CD8^+^ T cells in the liver [47, 48].

### Chimeric *P*. *berghei* parasites expressing *P*. *falciparum* proteins and analysis of sporozoite infectivity

For assessing protective efficacy of the *P*. *falciparum* vaccine candidates in mice, we generated chimeric rodent *P*. *berghei* ANKA parasites expressing the *P*. *falciparum* antigens. The fourteen genes encoding the *P*. *falciparum* antigens (Table 1, S1 Table) were introduced as an additional copy gene into the *p230p* or *s1* neutral loci of the *P*. *berghei* genome by GIMO transfection as described [25, 49–51]. All genes were placed under control of the strong *P*. *berghei uis4* promoter, that drives gene expression specifically in sporozoites and liver stages [25, 52]. Twelve DNA constructs, which also contained a GFP-Luciferase reporter gene for determination of parasite liver loads in live mice, were introduced into the neutral *p230p* locus and two constructs into the neutral *s1* locus [24, 53, 54] (S1 and S2 Figs). The details of the DNA constructs and the different chimeric parasite lines generated are shown in S1 Table. Correct integration of the *P*. *falciparum* antigen expression cassette into the genome of cloned chimeric *P*. *berghei* lines was confirmed by diagnostic Southern analysis of chromosomes separated by pulsed-field gel electrophoresis and diagnostic PCR (S1 and S2 Figs). All chimeric parasites showed normal asexual blood stage multiplication in mice and produced sporozoites comparable to wild type (WT) *P*. *berghei* parasites in *An*. *stephensi* mosquitoes (S2 Table).

**Fig 1:**
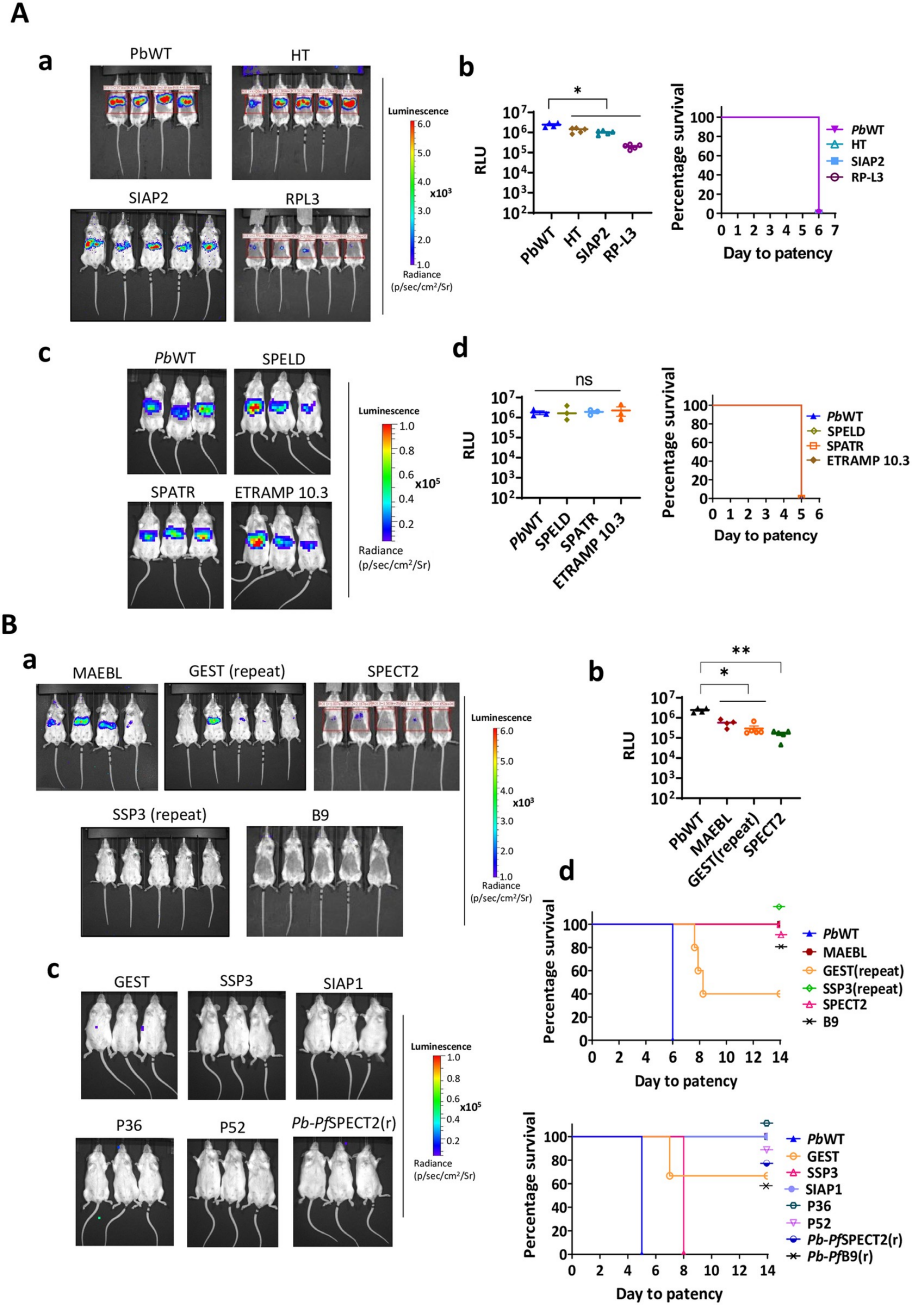
Sporozoite infectivity of chimeric *P*. *berghei* lines expressing *P*. *falciparum* proteins. Sporozoite infectivity is determined by measuring parasite liver load by *in vivo* imaging of luciferase-expressing liver stages at 44 hours post injection (hpi) of either 1x10^3^ or 1x10^4^ sporozoites and by measuring the day to blood stage patency, i.e. the day to reach 1% parasitemia. The Kaplan-Meier curves illustrate the prepatent period. Data shown from groups of 3–5 mice. (**A**) Chimeric *P*. *berghei* lines that showed WT-like infectivity in mice. **a**) Representative *in vivo* imaging (IVIS) of bioluminescence at 44 hpi shown in the livers of OF1 mice infected with 1x10^3^ chimeric *P*. *berghei* sporozoites of HT, SIAP2, and RPL3. **b**) Parasite liver loads in mice infected with chimeric *P*. *berghei* sporozoites of HT, SIAP2 and RPL3 expressed as relative luminescence units (RLU). Significance of RLU values (Mann-Whitney test): HT—0.0159, SIAP2–0.0159 and RPL3–0.0159. **c**) The Kaplan-Meier curve showing the prepatent period of HT, SIAP2 and RPL3 chimeric sporozoites. Significance values of day to patency [Log-Rank (Mantel-Cox) test]: n.s. **d**) Representative *in vivo* imaging (IVIS) of bioluminescence at 44 hpi shown in the livers of OF1 mice infected with 1x10^4^ chimeric *P*. *berghei* sporozoites of SPELD, SPATR and ETRAMP10.3. **e**) Parasite liver loads in mice infected with chimeric *P*. *berghei* sporozoites of SPELD, SPATR and ETRAMP10.3 expressed as relative luminescence units (RLU). Significance of RLU values (Mann-Whitney test): SPELD, SPATR and ETRAM10.3: not significant (n.s). **e**) The Kaplan-Meier curve showing the prepatent period of SPELD, SPATR and ETRAM10.3 chimeric sporozoites. Significance values of day to patency [Log-Rank (Mantel-Cox) test]: n.s. (**B**) Chimeric *P*. *berghei* lines that produced reduced or no sporozoite infectivity compared to WT sporozoites (MAEBL, GEST, SIAP1, SSP3, P36, P52, B9 and SPECT2). **a**) Representative *in vivo* imaging (IVIS) of bioluminescence at 44 hpi shown in the livers of OF1 mice infected with 1x10^3^ chimeric *P*. *berghei* sporozoites of MAEBL, GEST, SPECT2, SSP3 and B9. **b**) Parasite liver loads in mice infected with chimeric *P*. *berghei* sporozoites of MAEBL, GEST and SPECT2 expressed as relative luminescence units (RLU). Significance of RLU values (Mann-Whitney test): MAEBL—0.0286, GEST (rep)—0.0159 and SPECT2–0.0079. SIAP1, SSP3, P36, P52, B9: no luminescence signal. **c**) Representative *in vivo* imaging (IVIS) of bioluminescence at 44 hpi shown in the livers of OF1 mice infected with 1x10^4^ chimeric *P*. *berghei* sporozoites of GEST, SSP3, SIAP1, P36, P52 and *Pb-Pf*SPECT1(r). **d**) The Kaplan-Meier curve showing the prepatent period of MAEBL, GEST, SSP3, SPECT2 and B9 in mice infected with 1x10^3^ chimeric sporozoites (upper panel) and GEST, SSP3, SIAP1, P36, P52, *Pb-Pf*SPECT2(r) and *Pb-Pf*B9(r) in mice infected with 1x10^4^ chimeric sporozoites (lower panel). Significance values of day to patency [Log-Rank (Mantel-Cox) test]: GEST repeat (3/5 mice)– 0.0027, GEST (1/3 mice)– 0.0253 and SSP3 (3/3 mice)– 0.0253. The other chimeric lines did not initiate blood stage infections.

We next analyzed the infectivity of sporozoites of the chimeric lines by intravenous injection of 10^3^ or 10^4^ isolated salivary gland sporozoites in mice and analyzing parasite liver loads by *in vivo* imaging and prepatent period by analyzing blood stage parasitemia in Giemsa stained tail blood smears (Fig 1). Sporozoites of six of the fourteen chimeric lines, expressing HT, SPELD, SIAP2, SPATR, RP-L3 and ETRAMP10.3, exhibited *in vivo* infectivity in the range of WT *P*. *berghei* sporozoites (Fig 1).

Unexpectedly, sporozoites of eight chimeric parasite lines (GEST, SSP3, SIAP1, MAEBL, P36, P52, SPECT2 and B9) showed a strongly reduced infectivity as parasite liver loads were either absent or severely reduced after sporozoite infection. In addition, no blood stage infection was observed in mice infected with sporozoites of six of these eight lines and only for GEST and SSP3, a few mice became blood stage positive but with a 2–3 days prolonged prepatent period (Fig 1). These results suggest that expression of the *P*. *falciparum* ortholog is detrimental for sporozoite infectivity. To investigate the possibility that the reduced sporozoite infectivity resulted from an unexpected event during transfection and selection procedures, we performed independent transfections for GEST and SSP3. Also sporozoites from these independent chimeric lines demonstrated a reduced infectivity (Fig 1), indicating a negative impact of the *P*. *falciparum* proteins on sporozoite infectivity.

Another possibility is that reduced sporozoite infectivity results from the level and timing of expression of the *P*. *falciparum* proteins as in all chimeric lines the *P*. *falciparum* genes are under control of the strong *uis4* promoter and not placed under the promoter of its *P*. *berghei* ortholog. For two proteins, B9 and SPECT2, we therefore generated also so-called replacement lines where we replaced the endogenous *P*. *berghei b9* or *spect2* genes with the *P*. *falciparum* orthologs, resulting in expression of the *P*. *falciparum b9* or *spect2* under control of the orthologous *P*. *berghei* promoters (S3 Fig, S1 Table). Correct replacement of the *P*. *berghei* genes by the *P*. *falciparum* genes in the genome of the cloned replacement lines *Pb-Pfb9*(r) and *Pb-Pfspect2*(r) was confirmed by diagnostic Southern analysis of chromosomes separated by pulsed-field gel electrophoresis and diagnostic PCR (S3 Fig). Parasites of both replacement lines showed normal asexual blood stage multiplication in mice and produced sporozoites comparable to wild type (WT) *P*. *berghei* parasites in *An*. *stephensi* mosquitoes (S2 Table). However, sporozoites of both lines showed a strongly reduced infectivity as shown by reduced parasite liver loads and absence of blood stage infection in mice (Fig 1). The reduced infectivity of sporozoites in both the ‘additional copy’ and ‘replacement’ chimeric parasites indicates that the decreased sporozoite infectivity in the ‘additional copy’ parasites does not result from aberrant timing and level of expression due to the *uis4* promoter.

### Design of six ChAd/MVA viral vectored vaccines and immunization protocol

**Fig 2:**
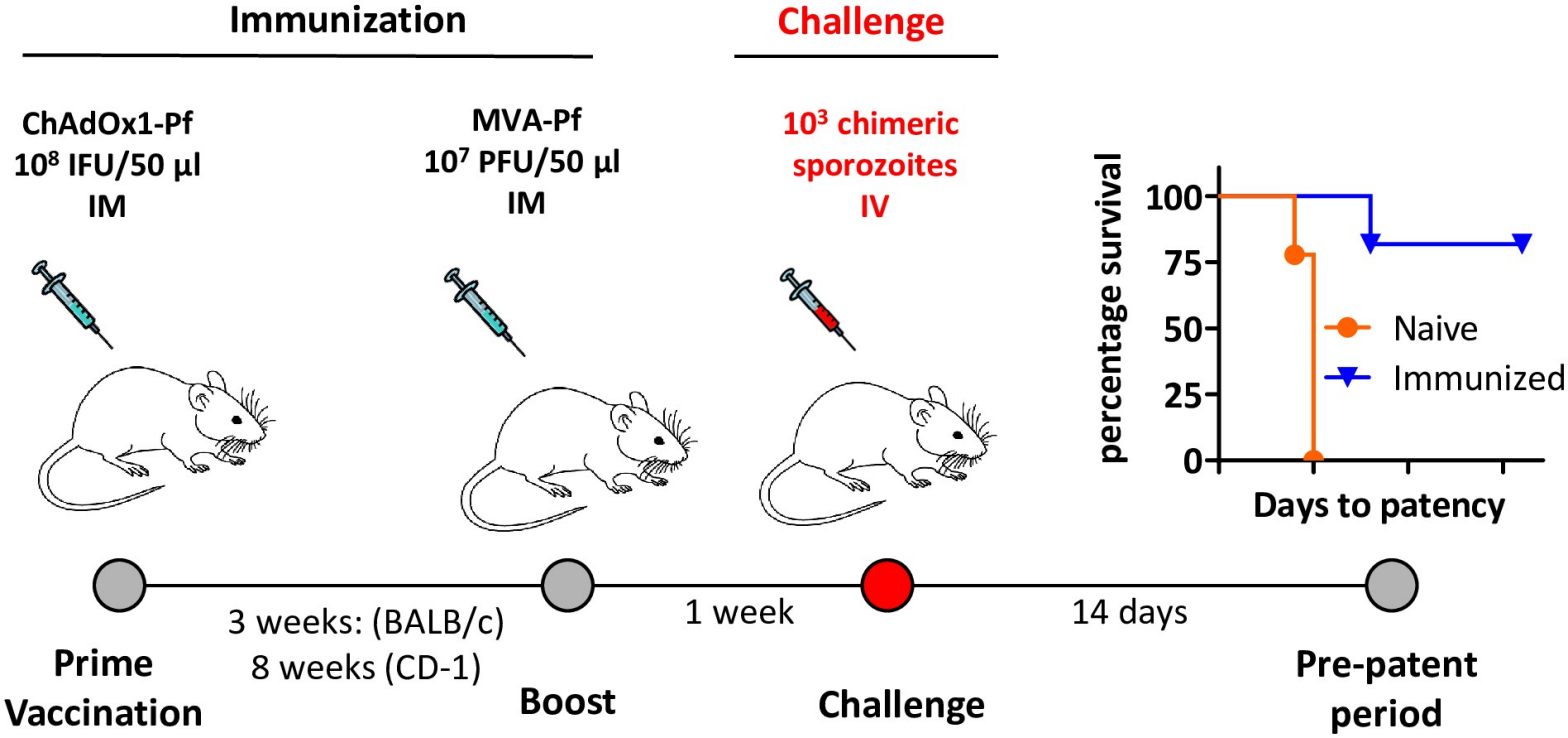
Schematic representation of the immunization and challenge protocol. BALB/c and CD-1 mice were immunized with ChAd63- and MVA viral-vectored *P*. *falciparum* proteins using a prime boost protocol followed by challenge with 1x10^3^ chimeric *P*. *berghei* sporozoites expressing *P*. *falciparum* proteins. The plot on the right shows visualization of the number of naïve or immunized mice that show no or protected immunity (either no blood stage infection or delay in the pre-patency) after challenge with the chimeric parasite expressing the cognate antigen. Protective immunity is determined by measuring the day to blood stage patency *i*.*e*., the day to reach 1% parasitemia.

Using established methods [25] we generated viral-vectored ChAd63/ChAdOx1 and MVA vaccines for the six *P*. *falciparum* proteins (HT, SPELD, SIAP2, SPATR, RPL3 and ETRAMP10.3) for which we were able to create chimeric *P*. *berghei* parasites that produced infectious sporozoites. The gene sequences of the candidate antigens in the viral vectors were based on the NF54 *P*. *falciparum* sequence from PlasmoDB (PlasmoDB v.46, www.plasmodb.org). The following minor modifications to the antigen sequence were made, i) the tissue plasminogen activator (tPA) leader sequence (GenBank Accession K03021) was added to the N-terminus of the encoded protein to enhance antigen secretion, expression and immunogenicity [55] and ii) all sequences were optimized to mammalian codon usage bias for expression in human cells. All genes were introduced under control of the modified human cytomegalovirus immediate-early promoter in the ChAd vector and under control of the p7.5 promoter in the MVA vector [25]. BALB/c mice and CD-1 mice were immunized with the ChAd63- and MVA-expressing *P*. *falciparum* antigens using the standard heterologous ChAd-MVA prime-boost intramuscular (i.m.) vaccination [25] regimen with a three week interval for BALB/c mice and 8 week interval for CD1 mice (Fig 2). A dose of 1x10^8^ infectious units (ifu) of ChAd63 or ChAdOx1 and 1x10^7^ plaque forming units (pfu) of MVA was used for immunization of both BALB/c and CD1 mice.

### Protective efficacy of six *P*. *falciparum* antigens

**Fig 3:**
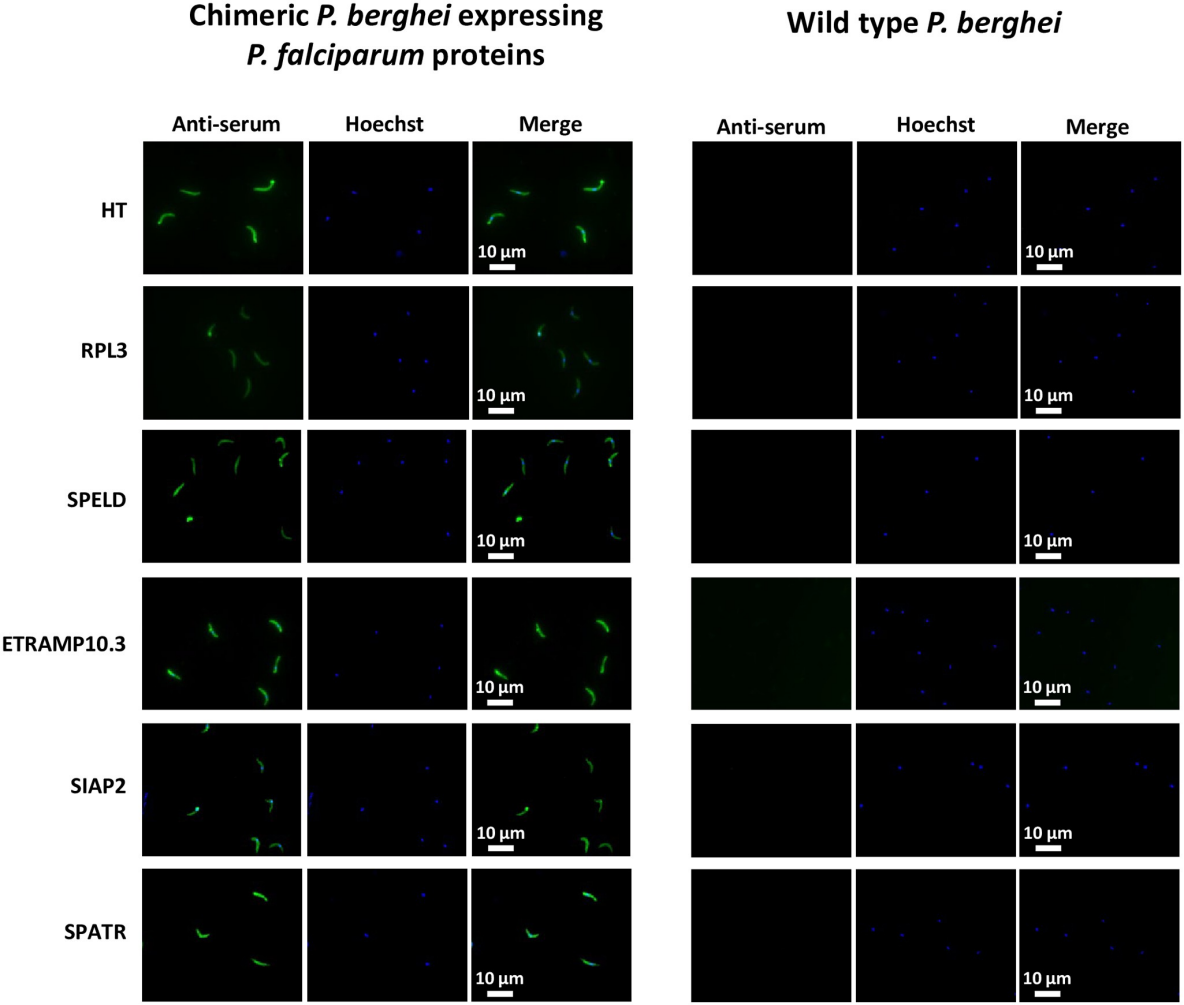
Expression of *P*. *falciparum* proteins in chimeric *P*. *berghei* sporozoites. Immunofluorescence analysis demonstrating *P*. *falciparum* protein expression in chimeric *P*. *berghei* sporozoites. Salivary-gland sporozoites of the different chimeric lines were stained with sera from the corresponding vaccinated mice (Alexa Fluor 488, green) and Hoechst-33342 (blue; nuclear staining). As a control, wild-type (WT) *P*. *berghei* sporozoites were stained with the same anti-sera. Merged images of the different channels are shown for both chimeric and WT *P*. *berghei* sporozoite stained images.

**Fig 4:**
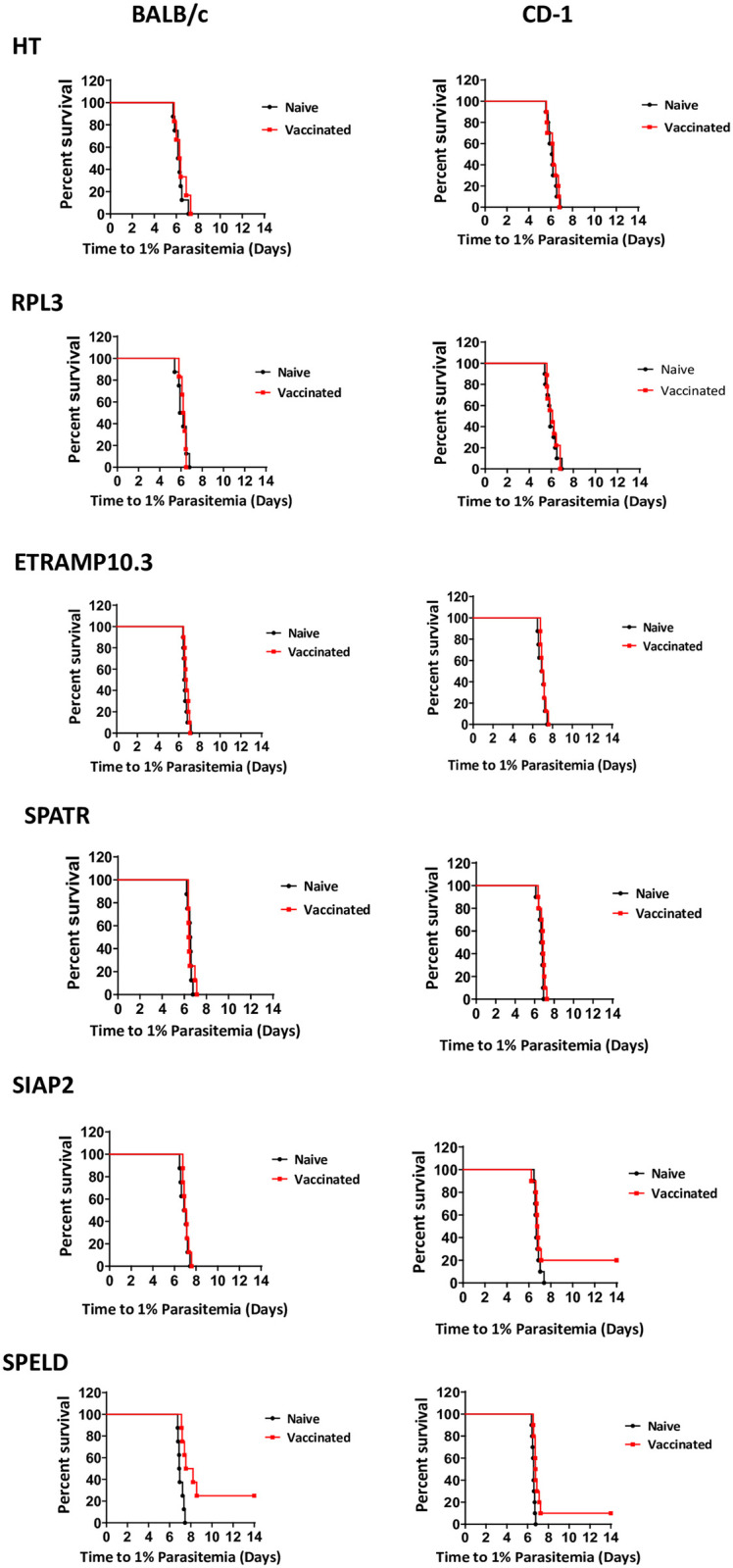
Protective immunity of viral-vectored *P*. *falciparum* antigens. Protective immunity in mice immunized with ChAd63- and MVA viral-vectored *P*. *falciparum* proteins as determined by the (delay in) the day to blood stage patency, i.e. the day to reach 1% parasitemia after challenge with chimeric *P*. *berghei* sporozoites expressing the *P*. *falciparum* proteins. Eight immunized and eight naïve BALB/c mice and ten immunized and ten naïve CD-1 mice were challenged with 10^3^ chimeric sporozoites by intravenous injection. The Kaplan-Meier curves illustrate the time to 1% parasitaemia and statistical significance between the survival curves was assessed using the Log-Rank (Mantel-Cox) Test (group size of inbred BALB/c mice: n = 6–8; group size of CD-1 outbred mice: n = 9–10. (a) PfHT p = 0.48 (BALB/c) and p = 0.77 (CD-1), (b) PfRPL3 p = 0.43 (BALB/c) and p = 0.86 (CD-1), (c) PfSPELD p = 0.003 (BALB/c) and p = 0.004 (CD1), (d) PfETRAMP10.3 p = 0.14 (BALB/c) and p = 0.38 (CD-1), (e) PfSIAP2 p = 0.48 (BALB/c) and p = 0.20 (CD-1), and (f) PfSPATR p = 0.73 (BALB/c) and p = 0.25 (CD-1).

We first analyzed expression of the six *P*. *falciparum* proteins (HT, SPELD, SIAP2, SPATR, RPL3 and ETRAMP10.3) in sporozoites of the six chimeric *P*. *berghei* parasites producing infectious sporozoites. Expression of all six *P*. *falciparum* antigens was confirmed by immunofluorescence assay analysis of fixed chimeric sporozoites incubated with serum from the vaccinated mice (Fig 3). Next, the protective efficacy of the six proteins was determined by challenge of the immunized BALB/c mice (n = 8) and CD-1 mice (n = 10) with 10^3^ chimeric sporozoites 1 week after the MVA boost. We challenged BALB/c and CD-1 mice in order to analyze the efficacy in both inbred (BALB/c) and outbred mice (CD-1). In previous studies using the same immunization and challenge approach to identity putative pre-erythrocytic vaccine candidate antigens we have found that in BALB/c mice a detectable cellular immune response was generated to all antigens and because these responses were more consistent than in C57BL/6 mice. We also conducted the same experiments in an outbred strain of mice, CD-1, in order to limit the effect of MHC restriction and immunodominance that can be observed in inbred strains of mice [25, 56]. Protective immunity is determined by measuring the prepatent period of blood stage infection (i.e. delay in the time to 1% parasitemia) (Fig 4) and for all challenge experiments, statistically significant efficacy (sterile protection or delay in prepatent period) was assessed using the Log-Rank (Mantel-Cox) Test. Vaccination with four of the six antigens (HT, RPL3, ETRAMP10.3 or SPATR) did neither induce sterile protection nor a statistically significant delay in the time to 1% parasitemia in both BALB/c and CD-1 mice. Vaccination with SIAP2 did not induce sterile protection in BALB/c mice (8 mice) but protected 2 out of 10 of the CD-1 mice. However, no significant delay in the time to 1% parasitemia was observed in both BALB/c and the unprotected CD-1 mice. Vaccination with SPELD protected 2 of the 8 BALB/c mice and 1 of the 10 CD-1 mice. The remaining mice had a significant delay in the time to 1% parasitemia, with a median delay of 0.55 days (p = 0.003) and 0.18 days (p = 0.0036) in BALB/c and CD-1 mice, respectively (Fig 4).

## Discussion

In this study we evaluated protective immunity in mice of six pre-erythrocytic proteins (SPELD, ETRAMP10.3, SIAP2, SPATR, HT, RPL3) delivered by a well-established prime-boost protocol using ChAd63 and MVA viral vectors [8]. Although SPELD induced some level of sterile protection (10–25% protected mice), none of the six proteins induced improved levels of protection compared to previously tested similar viral-vectored vaccines expressing CS, TRAP, LSAP2, or LSA1 [25]. These four proteins showed higher levels of sterile protection (30–80%) when analyzed using the same approach, i.e. viral vectored immunization of mice followed by challenge with chimeric rodent parasites that expressed the *P*. *falciparum* protein in sporozoites and liver (under control of the *uis4* promoter). It was shown that sterile protection obtained with LSAP2 and LSA1 was dependent on the presence of CD8+ T cells [25, 26]. A prime-boost regimen using viral vectored proteins has so far proved to be the most adept at inducing high-magnitude cellular immunity [57]. Viral-vectored vaccines expressing LSA1 and LSAP2 are currently undergoing clinical evaluation. Similar chimeric rodent parasites have also been used to analyze the effect of combining two viral vectored vaccines expressing the pre-erythrocytic *P*. *falciparum* antigens TRAP and UIS3 on protective immunity [54].

These earlier observations demonstrate that chimeric rodent parasites expressing *P*. *falciparum* proteins using the *uis4* promoter, can be recognized by immune responses induced in mice by viral-vectored proteins. Therefore, the low immunogenicity of the six proteins tested in this study may indicate an inferior intrinsic immunogenicity resulting from the lack of appropriate T-cell epitopes recognized by the immune system of BALB/C or CD-1 mice. In addition, viral-vectored expression of the proteins might result in the loss of conformational epitopes, resulting in the lack of inducing protective humoral immune responses targeting the selected proteins. However, several additional factors associated with the use of the chimeric mouse malaria models may explain the low protective immunity of these proteins. In all six ‘additional copy’ chimeric *P*. *berghei* parasites, the *P*. *falciparum* antigen is under control of the *P*. *berghei uis4* promoter. Whilst this places all antigens on an even level in terms of expression, the timing of expression in combination with structural differences between proteins may result in a different cellular location and exposure to the immune system compared to the proteins expressed from their endogenous promoters. It has been shown previously that differences in timing of expression of a protein in transgenic parasites can affect its cellular location [58]. It is known that the *usi4* promoter can drive expression of various proteins in transgenic sporozoites and liver stages that locate in the cytoplasm of these stages [26, 52, 54, 59]. For the surface protein CSP it has been demonstrated that *uis4*-driven expression of *P*. *falciparum* CSP also results in correct localization in chimeric parasites, i.e. at the surface of sporozoites [15]. For the proteins HT, SPELD, SIAP2 and SPATR that were analyzed in this study, evidence exists for a surface location in wild type sporozoites; however, we have not determined their location in detail in the chimeric parasites. It is therefore possible that wrong localization of the *P*. *falciparum* proteins in the chimeric parasites results in inaccessibility of these proteins to the appropriate (T cell-based) immune responses leading to the observed lack of parasite clearance and protection. However, the low immunogenicity might also be explained by the small numbers of MHC-restricted epitopes in mouse strain and marked immunodominance, which is less prominent in human populations [56]. It is known that specifically in inbred mice CD8(+) T cells respond to only a minute fraction of the potential peptide determinants, bound to MHC class 1 molecules, encoded by genomes of pathogens. For example, in viral infections, immunogenic determinants can be ordered into highly reproducible hierarchies based on the magnitude of cognate CD8(+) T cell responses, which is termed immunodominance. We have used in this study outbred CD-1 mice which may possibly reflect more accurately what may be seen in human studies but it remains possible that the proteins that were not protective in BALB/c and CD-1 mice may still be immunogenic in human populations. In our study we have chosen to only screen the proteins by measuring protective immunity and not analyzing or quantifying the T-cell or humoral responses induced in the mice by the viral vectored proteins. Our approach therefore excludes drawing conclusions about the relationships between immune responses induced by the viral vectored proteins and the observed lack of protection. In addition, viral-vectored expression of the proteins might result in the loss of conformational epitopes, resulting in the lack of inducing protective humoral immune responses targeting the selected proteins.

Unexpectedly, sporozoites of 8 of the 14 chimeric parasites showed a significant reduction of their infectivity. We had not observed this in our previous studies where we expressed different heterologous proteins, including *P*. *falciparum* proteins, in *P*. *berghei* sporozoites using the *uis4* promoter. By creation of additional, independent chimeric parasite lines for two proteins, GEST and SSP3, we provide evidence that reduced sporozoite infectivity is not the result from an unexpected event during transfection and selection procedures. The chimeric sporozoites from these independent chimeric lines also showed reduced sporozoite infectivity. These observations suggest that expression of the *P*. *falciparum* ortholog is detrimental for sporozoite infectivity. It is possible that the reduced infectivity results from the wrong timing and high level expression of the *P*. *falciparum* proteins using the strong *uis4* promoter. However, when we created for two of these proteins, B9 and SPECT2, replacement chimeric lines, we also observed reduced infectivity. In these replacement lines the endogenous *P*. *berghei b9* or *spect2* genes are exchanged with the *P*. *falciparum* orthologs, resulting in expression of the *P*. *falciparum b9* or *spect2* under control of the orthologous *P*. *berghei* promoters. These observations indicate that reduced sporozoite infectivity of the ‘additional copy’ chimeric lines does not result from aberrant timing and level of expression. Interestingly, most of the chimeric lines with reduced sporozoite infectivity express proteins with a role in hepatocyte cell traversal or in invasion and establishment of the parasitophorous vacuole in hepatocytes (GEST, SSP3, MAEBL, P36, P52, SPECT2, B9). It is known that *P*. *falciparum* sporozoites do not invade mouse hepatocytes and therefore it is possible that the presence of these *P*. *falciparum* proteins in sporozoites negatively affect traversal, invasion or early establishment in mouse hepatocytes. This is supported by studies showing that sporozoite infectivity is also reduced in chimeric *P*. *berghei* replacement lines in which the *P*. *berghei* gene has been exchanged with human ortholog. This has been reported for the PV proteins P36 and P52 [42] and for SPECT2 and B9 (this study) where sporozoites of chimeric replacement lines failed to establish a liver infection. Combined these observation demonstrate that several *P*. *falciparum* proteins cannot complement the function of the *P*. *berghei* orthologs during liver infection and indicate that expression of these *P*. *falciparum* proteins from an additional copy gene can have a detrimental effect on sporozoite infectivity. Therefore, chimeric rodent malaria models are unsuitable for these *P*. *falciparum* proteins to evaluate protective immunogenicity in immunization-challenge studies.

Although we could not demonstrate protective immunity with the six chimeric lines (SPELD, ETRAMP10.3, SIAP2, SPATR, HT, RP-L3) that produced infective sporozoites, these chimeric lines might be helpful to evaluate protective immunity induced by vaccine delivery platforms other than viral vectored proteins. Viral vectored proteins induce mainly cellular immunity while other platforms may induce humoral immune responses. For example, it has been shown that antibodies raised against SPATR blocked sporozoites invasion of cultured hepatocytes [28] and antibodies against SIAP2 reduced sporozoite cell traversal and infection of cultured hepatocytes [29, 33]. Chimeric rodent parasites expressing *P*. *falciparum* or *P*. *vivax* have been used already to evaluate protective immune responses induced by novel mRNA and virus-like particle (VLP) vaccine delivery systems [60, 61] and are useful tools to assess the efficacy of other malaria vaccine delivery systems.

## Materials and methods

### Experimental animals and P. berghei reference lines

In the Leiden University Medical Center female OF1 mice (6–7 weeks; Charles River, NL) were used and all animal experiments were granted with a licence by Competent Authority after an advise on the ethical evaluation by the Animal Experiments Committee Leiden (AVD1160020171625). All experiments were performed in accordance with the Experiments on Animals Act (Wod, 2014), the applicable legislation in the Netherlands in accordance with the European guidelines (EU directive no. 2010/63/EU) regarding the protection of animals used for scientific purposes. All experiments were executed in a licenced establishment for the use of experimental animals, Mice were housed in individually ventilated cages furnished with autoclaved aspen woodchip, fun tunnel, wood chew block and nestlets at 21 ± 2°C under a 12:12 hr light-dark cycle at a relative humidity of 55 ± 10%.

All animals and procedures in the University of Oxford were performed in accordance with the terms of the UK Home Office Animals Act Project License. Procedures were approved by the University of Oxford Animal Care and Ethical Review Committee (PPL P9804B4F1). Research staff involved in handling animals ha*ve* undergone special training accredited to Federation of European Laboratory Animal Science Associations.

All experiments involving generation of chimeric parasite lines, phenotype and protective analyses were performed using highly standardized and approved protocols that have been developed to reduce the number of animals and minimize suffering and distress. In all experiments mice were killed at a parasitemia of 2–5% before malaria-associated symptoms occur. Mice were killed either by cardiac puncture (under isoflurane anesthesia) or CO_2_.

Humane endpoints: The animals/body condition was thoroughly examined daily. Animals will be humanely sacrificed in case the following defined end points are reached: visible pain (abnormal posture and/or movement), abnormal behavior (isolation, abnormal reaction to stimuli, no food and water intake). If distress of the animals is observed by the animal caretakers, this will be reported to the investigators and according to the aforementioned criteria, the animals will be taken out of the experiment and euthanized. In all experiments no mice were euthanized before termination of the experiment and no mice died before meeting criteria for euthanasia.

The following numbers of mice were used for the different experiments: i) Generation of chimeric parasite lines: 672 (32 mice per line); ii) Sporozoite production: 58 (2 mice per line); iii) Fitness analyses of chimeric parasite lines: 73 (3–6 mice per line); Protective efficacy: 115 CD1 and 95 BALB/c. The duration of all animal experiments was between 5 to 15 days. Two *P*. *berghei* ANKA reference parasite lines were used: i) 1596cl1 (230p-GIMO_PbANKA_; RMgm-687, www.pberghei.eu) which contains a positive-negative selectable marker (SM; human *dihydrofolate reductase*:: *yeast cytosine deaminase and uridyl phosphoribosyl transferase* (h*dhfr*::*yfcu*)) cassette integrated into the silent *230p* gene locus (PBANKA_0306000) [50], ii) the reporter line 676m1cl1 (ANKA-GFP-Luc_con_; mutant RMgm-29; www.pberghei.eu) which contains a GFP-luciferase fusion gene under the control of constitutive eef1α promoter integrated into the *230p* gene locus [62]. In addition, reporter-free *P*. *berghei* ANKA *Pb*ΔB9-GIMO was used (1309cl1; RMgm-932; www.pberghei.eu) [40].

### Generation of chimeric P. berghei parasites

Twelve ‘additional copy’ chimeric *P*. *berghei* ANKA parasites expressing twelve different *P*. *falciparum* proteins (Table 1) were generated by replacing the positive negative drug selectable marker (SM) cassette in the *230p* (PBANKA_0306000) locus of 1596cl1 parasites with the transgene expression cassette by ‘gene insertion/marker out’ (GIMO) technology [24, 25] (S1 Fig). This was achieved by first generating the basic plasmid, pL2281, based on the basic *230p* targeting plasmid pL0043 [63]. The *Pbuis4* 5’*utr* (1.5 Kb) and 3’*utr* (1 Kb) sequences were amplified using the primers 7169/7170 and 7171/7172, respectively, and introduced into pL0043 using the restriction sites PstI/NotI and EcoRV/KpnI, respectively, to obtain pL2005. Next, a *gfp*::*luc* fusion reporter gene under the constitutive *Pbeef1a* promoter was amplified from the plasmid pL0027, (MRA-796, https://www.beiresources.org) using the primers 7291/7292, and introduced at the KpnI restriction site of pL0025 to obtain pL2281. The open reading frame (ORF) of the twelve *P*. *falciparum* genes were amplified from *P*. *falciparum* (NF54 strain) genomic DNA using primers described in S1 and S3 Tables and the ORFs were inserted into plasmid pL2281 in between the 5’*utr* and *3’utr* of *Pbuis4* using the restriction sites NotI and BsaBI to obtain the final constructs that contain both the *P*. *falciparum* gene expression cassette and the *gfp*::*luc* expression cassette (S1 Table). All constructs were linearized with SacII and introduced into 1596cl1 parasites using standard methods of GIMO-transfection [50]. Transfected parasites were selected by applying negative selection by providing 5-fluorocytosine (5-FC) in the drinking water of the mice [24]. Negative selection results in the selection of chimeric parasites where the h*dhfr*::y*fcu* SM in the *230p* locus is replaced by the *P*. *falciparum* gene expression cassette and the *gfp*::*luc* expression cassette. All twelve selected chimeric parasites were cloned by the method of limiting dilution [64] and correct integration of the targeting constructs were analyzed by diagnostic PCR analysis of gDNA and Southern analysis of PFG-separated chromosomes as described [65]. See S1 Table for details of the chimeric lines and S3 Table for details of primers. Two ‘additional copy’ chimeric *P*. *berghei* ANKA parasites (expressing B9 and MAEBL; S1 Table) were generated by introducing the *P*. *falciparum* gene expression cassette into a neutral *Pbs1* gene (PBANKA_1206800) of 676m1cl1 parasites using the 2-step ‘gene insertion/marker out’ (GIMO) transfection protocol [50, 53] (S2 Fig). In the first step, a linear DNA construct (plasmid pL1928) that has a positive/negative h*dhfr*::*yfcu* SM cassette was introduced into the *Pbs1* gene locus to obtain *Pbs1* GIMO mother line 2149cl2 (RMgm-4434, www.pberghei.eu). Plasmid pL1928 was generated using the GIMO-plasmid pL0034 (MRA-849, www.beiresources.org) [50], containing the *hdhfr*::*yfcu* SM. This plasmid was used to insert both the *5’* (0.7 Kb) and *3’* gene (0.8 Kb) targeting regions (TR) of *Pbs1*. The *Pbs1* 5’*utr* was PCR amplified using primer pair 1003/1004 and ligated into pL0034 using ApaI and StuI restriction sites. Next, the *Pbs1 3*’*utr* was PCR amplified using the primer pair 1005/1006 and ligated using KasI and NotI restriction sites, resulting in plasmid pL1928 (primers described in S1 and S3 Tables). Plasmid pL1928 was linearized using KasI and ApaI restriction sites and transfected into 676m1cl1 parasites using GIMO-transfection [50]. Transfected parasites were selected in mice by applying positive selection by providing pyrimethamine in the drinking water [65]. Positive selection results in selection of transgenic parasites where the *s1* gene is replaced by the *hdhfr*::*yfcu* SM, resulting in line 2149. Parasites of line 2149 were cloned by limiting dilution and clone 2149cl2 (*Pbs1* GIMO) line was used for step 2. Correct integration of SM cassette into the *Pbs1* locus was confirmed by diagnostic PCR analysis of genomic DNA (gDNA) and Southern analysis of pulsed-field gel electrophoresis-separated chromosomes as described previously [65] (S2 Fig). In step 2, we replaced the *hdhfr*::*yfcu* SM in *Pbs1* GIMO parasites with the transgene expression cassette. This was achieved by first generating the plasmid, pL2046, by ligating the *5’utr* and *3’utr* sequences of *Pbuis4* from pL2005 into pL1928 using restriction sites KpnI and PstI to obtain pL2046. The ORFs of *b9* and *maebl* were amplified from *P*. *falciparum* (NF54 strain) genomic DNA using primers described in S1 and S3 Tables and inserted into plasmid pL2046 in between the 5’*utr* and *3’utr* of *Pbuis4* using the restriction sites NotI and BsaBI to obtain pL2041 and pL2289, respectively. Plasmid pL2041 was linearized using ApaI and KasI and pL2289 with HindIII and transfected in *Pbs1*-GIMO parasites using GIMO-transfection [50]. Transfected parasites were selected by applying negative selection providing 5-fluorocytosine (5-FC) in the drinking water of the mice [24]. Negative selection results in the selection of chimeric parasites where the h*dhfr*::y*fcu* SM in the *s1* locus is replaced by the *P*. *falciparum* gene expression cassette. The two selected chimeric parasite lines were cloned by the method of limiting dilution [64] and correct integration of the targeting constructs were analyzed by diagnostic PCR analysis of gDNA and Southern analysis of PFG-separated chromosomes [65] (S2 Fig). See S1 Table for details of the chimeric lines and S3 Table for details of primers.

The ‘replacement’ chimeric parasite line expressing *Pf*SPECT2 (PF3D7_0408700) was generated using the 2-step GIMO transfection protocol [50] (S3 Fig). In the first step, the *Pbspect2* ORF (PBANKA_1006300) was replaced with the positive-negative h*dhfr*::*yfcu* SM to create a *Pbspect2* deletion GIMO line (*PbΔspect2*). This was achieved by generating a DNA construct pL2318, that has 5’ and 3’ targeting regions of *Pbspect2* ligated in GIMO DNA construct pL0034 [50]. Plasmid pL2318 was linearized with ApaI/NotI and transfected into parasites of 676cl1 parasites using standard methods of transfection [65]. *PbΔspect2* parasites (line 3144) were selected by positive selection providing pyrimethamine in the drinking water of mice [65]. Transfected parasites were cloned by limiting dilution [64], and correct integration of the targeting constructs were analyzed by diagnostic PCR analysis of gDNA and Southern analysis of PFG-separated chromosomes as described [65] (S3 Fig). In the second step, we replaced the h*dhfr*::*yfcu* SM in *PbΔspect2* with the *Pfspect2* ORF by GIMO transfection to create a chimeric *Pb-Pfspect2*(r) replacement line. This was achieved by replacing the h*dfhr*::y*fcu* SM in pL2318 with the *Pfspect2* ORF sequence, generating plasmid pL2319. The *Pfspect2* ORF was amplified from *Pf*NF54 genomic DNA using the primers 1289 and 1290, digested with SacII/KpnI and ligated into vector pL2318 to obtain pL2319. The construct was linearized using ApaI and NotI restriction enzymes and transfected into *PbΔspect2* (line 3144cl3) by standard methods of GIMO transfection [50]. Transfected parasites (line 3162) were selected in mice by applying negative selection by providing 5-fluorocytosine (5-FC) in the drinking water [24]. Negative selection results in the selection of chimeric parasites where the h*dhfr*::y*fcu* SM locus is replaced by *Pfspect2* ORF Selected chimeric parasites were cloned by limiting dilution and correct integration of the construct into the genome of *Pb-Pfspect2*(r) (line 3162cl2) was analyzed by diagnostic PCR analysis of gDNA and Southern analysis of PFG-separated chromosomes as described previously [65] (S3 Fig). See S1 Table for details of the chimeric line and S3 Table for details of primers.

The ‘replacement’ chimeric parasite line expressing *Pf*B9 (PF3D7_0317100) was generated by introducing the *Pfb9* ORF into the *b9* locus (PBANKA_0808100) of *Pb*ΔB9-GIMO parasites using GIMO transfection [40, 50]. The h*dhfr*::*yfcu* SM in *Pb*B9 GIMO line (1309cl1) was replaced with the *Pfb9* ORF by GIMO transfection to create a chimeric *Pb-Pfb9*(r) replacement line. This was achieved by replacing the mCherry ORF with the *Pfb9* ORF, amplified from *Pf*NF54 genomic DNA using the primers 7225/7226, into plasmid pL1695 [40] using the restriction sites BamHI and SgrAI to obtain pL1989. Plasmid pL1989 was linearized using AflII and SacI restriction enzymes and transfected in *PbΔ*B9-GIMO parasites using GIMO-transfection [50]. Transfected parasites were selected by applying negative selection providing 5-fluorocytosine (5-FC) in the drinking water of the mice [24]. Negative selection results in the selection of chimeric parasites where the h*dhfr*::y*fcu* SM locus is replaced by *Pfb9* ORF. Selected chimeric parasites were cloned by limiting dilution and correct integration of the construct into the genome of *Pb-Pfb9*(r) (line 2355cl1) was analyzed by diagnostic PCR analysis of gDNA and Southern analysis of PFG-separated chromosomes as described previously [65] (S3 Fig). See S1 Table for details of the chimeric lines and S3 Table for details of primers.

### Phenotype analysis of the chimeric P. berghei parasites

The asexual multiplication rate of different chimeric parasites *in vivo* was determined during the cloning procedure of the chimeric parasites [66]. For mosquito transmission experiments, female *An*. *stephensi* mosquitoes were fed on mice infected with either wild-type or chimeric parasites and oocyst and sporozoite production was monitored in infected mosquitoes as described [67]. Salivary gland sporozoites (sg-spz) were isolated by manual dissection of mosquitoes on day 21 post feeding and counted in a Bürker cell counter using phase-contrast microscopy as described [49]. The *in vivo* infectivity of sporozoites was determined by analysis of parasite liver load and by determination of prepatent period to blood stage infection in OF1 mice after intravenous injection of 1x10^4^ (at LUMC) or 1x10^3^ at The Jenner Institute) salivary gland sporozoites. Parasite liver loads were visualized and quantified by real time *in vivo* imaging at 44 h post infection [49]. Blood stage infections were monitored in Giemsa stained tail blood smears. The prepatent period is defined as the time to reach 1% parasitemia [25].

*Immunofluorescence analysis of P*. *falciparum protein expression in chimeric P*. *berghei sporozoites* Expression of the *P*. *falciparum* proteins in chimeric parasites was analysed by immunofluorescence assay. Sporozoites were loaded onto glass slides and permeabilized with 0.25% triton X 100 after fixing with 4% paraformaldehyde. The slides were then blocked with 10% FCS and 1% BSA in PBS before the addition of serum from vaccinated mice. Bound IgG was detected with goat anti-mouse IgG-Alexa Fluor 488 (Life Technologies) and nuclear DNA stained with 2% Hoechst-33342. Slides were mounted with Fluorescence Mounting Medium (Dako, Denmark) and viewed under a Leica DMI-300B microscope.

### Design of ChAd/MVA viral vectored vaccines and immunization protocol

Viral-vectored ChAd63- and MVA vaccines for the six *P*. *falciparum* proteins (HT, SPELD, SIAP2, SPATR, RP-L3 and ETRAMP10.3) were generated as described by Longely et al [25] with some minor modifications. The gene sequences of the candidate antigens in the viral vectors were based on the NF54 *P*. *falciparum* sequence from PlasmoDB (PlasmoDB v.46, www.plasmodb.org). The following minor modifications to the antigen sequence were made, i) the tissue plasminogen activator (tPA) leader sequence (GenBank Accession K03021) was added to the N-terminus of the encoded protein to enhance antigen secretion, expression and immunogenicity [55] and ii) all sequences were optimized to mammalian codon usage bias for expression in human cells. All genes were introduced under control of the modified human cytomegalovirus immediate-early promoter in the ChAd vector and under control of the p7.5 promoter in the MVA vector [25].

#### Immunization of mice

Mice were immunized (prime immunization) by intramuscular injection into the musculus tibialis with 50 μl endotoxin free D-PBS containing 1 × 10^8^ infectious units (ifu) of simian adenoviral vector 63 (ChAd63) encoding the *P*. *falciparum* gene. At 3 or 8 weeks after priming, the mice were boosted with 1 × 10^7^ plaque forming units (pfu) MVA expressing the same *P*. *falciparum* gene. All recombinant ChAd63 and MVA viral vectors used throughout this study were generated at The Jenner Institute’s vector core facility.

### Protective efficacy

Immunized mice were challenged one week after the MVA boost by intravenous injection of 1x10^3^ salivary gland sporozoites. Protective efficacy was determined by analysis of parasite liver load and by determination of prepatent period to blood stage infection. Parasite liver loads were visualized and quantified by real time *in vivo* imaging at 44 h post infection [49]. Blood stage infections were monitored in Giemsa stained tail blood smears. The prepatent period is defined as the time to reach 1% parasitemia [25].

### Statistical analysis

GraphPad Prism version 8 (GraphPad, USA) was used for all analyses. Survival analyses is presented using Kaplan-Meier curves and significance tested for challenge studies was performed using the Log-Rank (Mantel-Cox) Test. For the median delay in pre-patency, statistical significance was assessed after removing the protected mice (absence of blood infection). The significance threshold was 0.05.

## Supporting information

S1 Fig Generation and genotyping chimeric *P*. *berghei* lines expressing *P*. *falciparum* proteins (additional copy lines; *230p* locus). **A**. Schematic representation of the introduction of the *P*. *falciparum* gene expression-cassettes by double cross-over integration into the *230p* locus of the *P*. *berghei* ANKA GIMO-*230p* mother line by GIMO-transfection. The *P*. *falciparum* gene is under control of the *P*. *berghei uis4* regulatory sequences (5’UTR and 3’UTR). The construct also contains a *gfp*::*luc* fusion reporter gene under the constitutive *Pbeef1a* promoter. Black arrows: location of PCR primers used for diagnostic PCR-analysis (panel **C**). The table shows the 14 chimeric parasite lines generated (see S1 Table for details). *orf*, open reading frame; int, integration. **B**. Genotype analysis of the 14 chimeric parasite lines parasites by Southern analysis of chromosomes (chr.) separated by pulsed-field gel electrophoresis to confirm integration of the DNA constructs in the GIMO locus (*230p* on chr. 3), shown as the absence of the h*dhfr*::y*fcu* selectable marker (SM) cassette in cloned chimeric parasites by hybridisation of chr. with the h*dhfr* probe. Chromosomes are also hybridized to a control probe recognising chr. 5. As an additional control (ctrl), parasite line 2117cl1 is used with the *hdhfr*::*yfcu* SM integrated into chr. 3. **C**. Diagnostic PCR analysis of the 14 chimeric parasite lines confirming correct integration of the *P*. *falciparum* antigen expression cassettes. Correct integration in all lines is shown by the presence of the *P*. *falciparum* gene coding sequence, absence of the h*dhfr*::y*fcu* SM, and the correct integration of the construct into the genome at both the 5’and 3’regions (5’ int and 3’ int). See panel **A** for the location of the primers. Primer details, sequences and the expected PCR product sizes are shown in S3 Table.
(PDF)

S2 Fig Generation and genotyping chimeric *P*. *berghei* lines expressing *P*. *falciparum* proteins (additional copy lines; *s1* locus). **A**. Schematic representation of the introduction of the *P*. *falciparum* expression-cassette (*b9/maebl*) by double cross-over integration into the *s1* locus of *P*. *berghei* ANKA GIMO-*s1* mother line by GIMO-transfection. The *P*. *falciparum* gene is under control of the *P*. *berghei uis4* regulatory sequences (5’UTR and 3’UTR). Black arrows: location of PCR primers used for diagnostic PCR-analysis (panel **B**). The table shows different chimeric parasites generated. *Orf*, open reading frame; int, integration. **B**. Left: Genotype analysis of chimeric parasite lines by Southern analysis of chromosomes (chr.) separated by pulsed-field gel electrophoresis (PFGE). The correct integration of construct in the chimeric lines was confirmed by showing the absence of the h*dhfr*::y*fcu* selectable marker (SM) cassette in cloned chimeric parasites by hybridisation of chr. with the h*dhfr* probe. Chromosomes are also hybridized to a control probe recognising chr. 5. As an additional control (ctrl), parasite line 2117cl1 is used with the h*dhfr*::y*fcu* SM integrated into chr. 3. Right: Diagnostic PCR analysis confirms the correct integration of the *P*. *falciparum b9* and *maebl* expression cassettes in the chimeric parasites. Correct integration is shown by the presence of the *Pfb9* or *Pfmaebl* orf, absence of the h*dhfr*::y*fcu* SM, and the correct integration of the construct into the genome at both the 5’ and 3’ regions (5’int and 3’int). See panel **A** for the location of the primers. Primer details, sequences and the expected PCR product sizes are shown in S3 Table.
(PDF)

S3 Fig Generation and genotyping chimeric *P*. *berghei* lines expressing *P*. *falciparum* proteins (replacement lines). **A**. Schematic representation of the *Pb230p* locus of the reference reporter *P*. *berghei* ANKA parasite 676cl1 which was used to generate the chimeric *Pb-Pfspect2*(r) parasite line (see **B**). This parental line contains a *gfp-luciferase* fusion reporter gene under the constitutive *Pbeef1a* promoter and is selectable marker (SM) free. The reporter-cassette is integrated into the neutral *230p* locus in chromosome 3. **B**. Schematic representation of the generation of the chimeric line *Pb-Pfspect2*(r) (line 3162cl2). First step: the GIMO deletion-construct (construct 1; pL2318) was used to replace the *Pbspect2* open reading frame (*orf*) with the positive/negative selectable marker (SM; h*dhfr*::*yfcu*) cassette, resulting in the generation of the *PbΔspect2* (line 3144cl3) after positive selection with pyrimethamine. Construct 1 targets the *Pbspect2* gene by double cross-over homologous recombination. After genotyping and confirmation of correct construct integration, this line was cloned by limiting dilution. Int, integration. Second step: The GIMO insertion construct (construct 2; pL2319) was used to replace the SM in the *PbΔspect2* GIMO line with the *Pfspect2 orf*, resulting the generation of line *Pb-Pfspect2*(r) (line 3162cl2) after negative (5-FC) selection. Construct 2 integrates by double cross-over homologous recombination using the same targeting regions employed in construct 1, resulting in the Introduction of the *Pfspect2 orf* under the control of *Pbspect2* regulatory sequences. Black arrows: number and location of primers used for diagnostic PCR (panel **C**). **C**. Left: Genotype analysis of *PbΔspect2* and *Pb-Pfspect2*(r) parasites by Southern analysis of chromosomes (chr.) separated by pulsed-field gel electrophoresis (PFGE). Hybridisation of PFG-separated chr. of *PbΔspect2* with a mixture of *hdhfr* and a probe specific for chr. 5 confirms integration of construct 1 into the *Pbspect2* gene on chr. 10 in *Pbspect2*-GIMO (line 18). The correct integration of construct 2 in *Pb-Pfspect2*(r) (line 19) was confirmed by showing the absence of the h*dhfr*::y*fcu* selectable marker (SM) cassette in cloned chimeric parasites by hybridisation of chr. with the *hdhfr* probe. Chromosomes are also hybridized to a control probe recognising chr. 5. As an additional control (ctrl), parasite line 2117cl1 is used with the *hdhfr*::*yfcu* SM integrated into chr. 3. Right: Diagnostic PCR analysis confirms the deletion of *Pbspect2* in *PbΔspect2*-GIMO and the correct integration of the *Pfspect2* expression cassette in *Pb-Pfspect2*(r). Correct integration is shown by the absence of *Pbspect2 orf* in *PbΔspect2*-GIMO, the absence of *hdhfr*::*yfcu* SM and the presence of the *Pfspect2 orf* in P*b-Pfspect2*(r) and the and the correct integration of the construct into the genome at both the 5’ and 3’ regions (5’int and 3’int; see **B** for primer numbers and locations). Primer details, sequences and the expected PCR product sizes are shown in S3 Table. **D**. Schematic representation of the generation of the chimeric line *Pb-Pfb9*(r). The GIMO insertion construct (pL1899) was used to replace the h*dhfr*::y*fcu* SM in the *PbΔb9* GIMO line (line 1309cl1) with the *Pfb9* open reading frame (*orf*), resulting the generation of line *Pb-Pfb9*(r) (line 2355cl1) after negative (5-FC) selection. Construct pL1989 integrates by double cross-over homologous recombination using *Pbb9* targeting regions, resulting in the Introduction of the *Pfb9 orf* under the control of *Pbb9* regulatory sequences. Black arrows: location and primer number used for diagnostic PCR (right panel of C). Black arrows: location of PCR primers used for the diagnostic PCR-analysis (panel **E**). Int, integration. **E**. Left: Genotype analysis of *Pb-Pfb9*(r) line (line 20) by Southern analysis of chromosomes (chr.) separated by pulsed-field gel electrophoresis (PFGE) (left) and diagnostic PCR analysis (right). The correct integration of construct in the chimeric lines was confirmed by showing the removal of the h*dhfr*::*yfcu* selectable marker (SM) cassette by hybridisation of chr. with the *hdhfr* probe. Chromosomes are also hybridized to a control probe recognising chr. 5. As an additional control (ctrl), parasite line 2117cl1 is used with the h*dhfr*::y*fcu* SM integrated into chr. 3. Right: Diagnostic PCR analysis confirms the correct integration of the *P*. *falciparum b9* expression cassette in *Pb-Pfb9*(r). Correct integration is shown by the presence of *Pfb9 orf*, absence of the *hdhfr*::*yfcu* SM, and the correct integration of the construct into the genome at both the 5’ and 3’ regions (5’int and 3’int; see **D** for primer numbers and locations). Primer details, sequences and the expected PCR product sizes are shown in S3 Table.
(PDF)

S4 Fig Generation and genotyping of the *s1*-GIMO *P*. *berghei* line. **A**. Schematic representation of the *Pb230p* locus of the reference reporter *P*. *berghei* ANKA parasite 676cl1 which was used to generate the chimeric *Pb-Pfspect2*(r) parasite line (see **B**). This parental line contains a *gfp-luciferase* fusion reporter gene under the constitutive *Pbeef1a* promoter and is selectable marker (SM) free. The reporter-cassette is integrated into the neutral *230p* locus in chromosome 3. **B**. Schematic representation of the generation of the *Pbs1* GIMO. The GIMO deletion-construct (pL1928) was used to replace the *Pbs1* coding sequence (CDS) with the positive/negative selectable marker (SM; h*dhfr*::*yfcu*) cassette, resulting in the generation of the *Pbs1* GIMO (line 2149cl2) after positive selection with pyrimethamine. The construct pL1928 targets the *Pbs1* gene by double cross-over homologous recombination. After genotyping and confirmation of correct construct integration, this line was cloned by limiting dilution. **C**. Left: Genotype analysis of *Pbs1* GIMO parasites by Southern analysis of chromosomes (chr.) separated by pulsed-field gel electrophoresis (PFGE) (left) and diagnostic PCR analysis (right). Hybridisation of PFG-separated chr. of *Pbs1* GIMO with a 3’ UTR *Pbdhfr/ts* probe. This probe recognizes the construct integrated into chr. 12, the endogenous *Pbdhfr/ts* gene at chromosome 7 and the *gfp-luciferase* reporter cassette at chr. 3. As an additional control (ctrl), parasite line 2117cl1 is used with a construct containing the 3’ UTR *Pbdhfr/ts* integrated into chr. 3. Diagnostic PCR analysis confirms the deletion of *Pbs1* in *Pbs1* GIMO. Correct integration is shown by the absence of *Pbs1 orf*, the presence of *hdhfr*::*yfcu* SM and the correct integration of the construct into the genome at both the 5’ and 3’ regions (5’int and 3’int; see **B** for primer numbers and locations). Expected PCR product sizes and the primer numbers are listed in the Table in the Figure and primers sequences are shown in S3 Table.
(PDF)

S5 Fig Autoradiographs of Southern analysis of chromosomes (chr.) separated by pulsed-field gel electrophoresis (PFGE) and pictures of agarose gels containing PCR fragments of chimeric *P*. *berghei* lines. **A**. names and line numbers of 20 transgenic parasite lines described in this study (see S1 Table for full details of the lines). **B**. Full length agarose gel images containing PCR fragments of the different chimeric *P*. *berghei* lines. (see S1–S4 Figs for description of the primers used for the different fragments). **C**. Autoradiographs of Southern analysis of chromosomes (chr.) separated by pulsed-field gel electrophoresis (PFGE). Red boxes indicate the different chimeric lines. See Table in panel **A** for the numbers of the chimeric lines. Ctrl: control line.
(PDF)

S1 Table Details of chimeric *P*. *berghei* lines generated. (XLSX)

S2 Table Sporozoite production of *P*. *berghei* chimeric lines. (XLSX)

S3 Table Primers used in the study. (DOCX)
