# Supplementary material for: Screening of viral-vectored P. falciparum pre-erythrocytic candidate vaccine antigens using chimeric rodent parasites
Source: PLoS One. 2021 Jul 12;16(7):e0254498. doi: 10.1371/journal.pone.0254498 (PMC8274855; doi:10.1371/journal.pone.0254498)

A

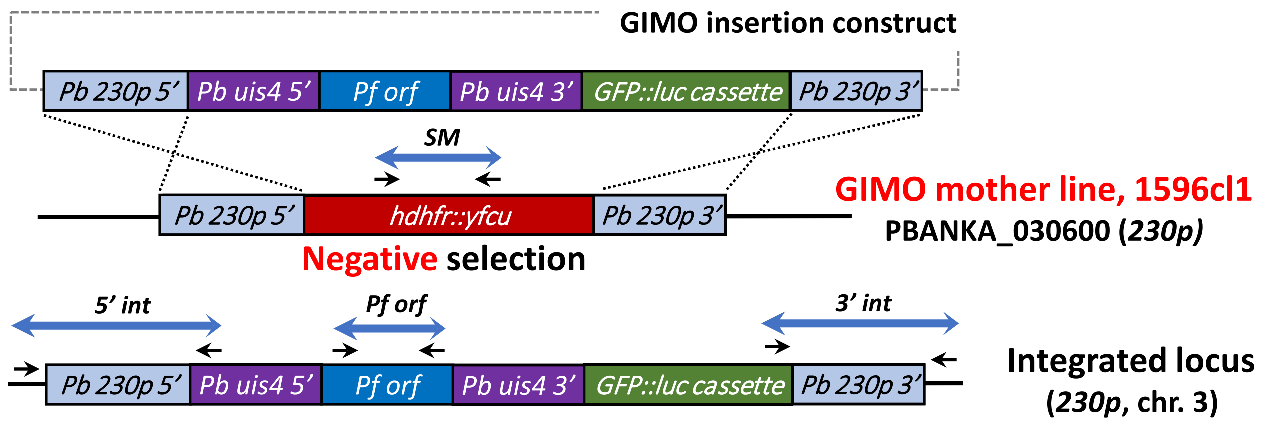

S.No	Chimeric parasite	Line #	S.No	Chimeric parasite name	Line #
1	HT@PbUIS4	2409cl4	8	SSP3@PbUIS4	3018cl1
2	RP-L3@PbUIS4	2411cl1	9	SIAP1@PbUIS4	2909cl4
3	SPELD@PbUIS4	2887cl1	10	SIAP2@PbUIS4	2911cl1
4	GEST@PbUIS4	2888cl4	11	SPATR@PbUIS4	3026cl1
5	GEST@PbUIS4	3015cl1	12	P36@PbUIS4	3029cl1
6	ETRAMP@PbUIS4	2891cl1	13	P52@PbUIS4	3032cl1
7	SSP3@PbUIS4	2895cl1	14	SPECT2@PbUIS4	3039cl1

B

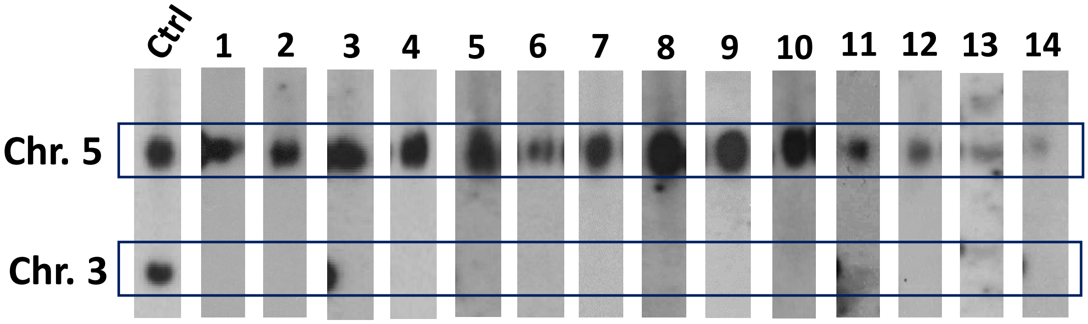

C

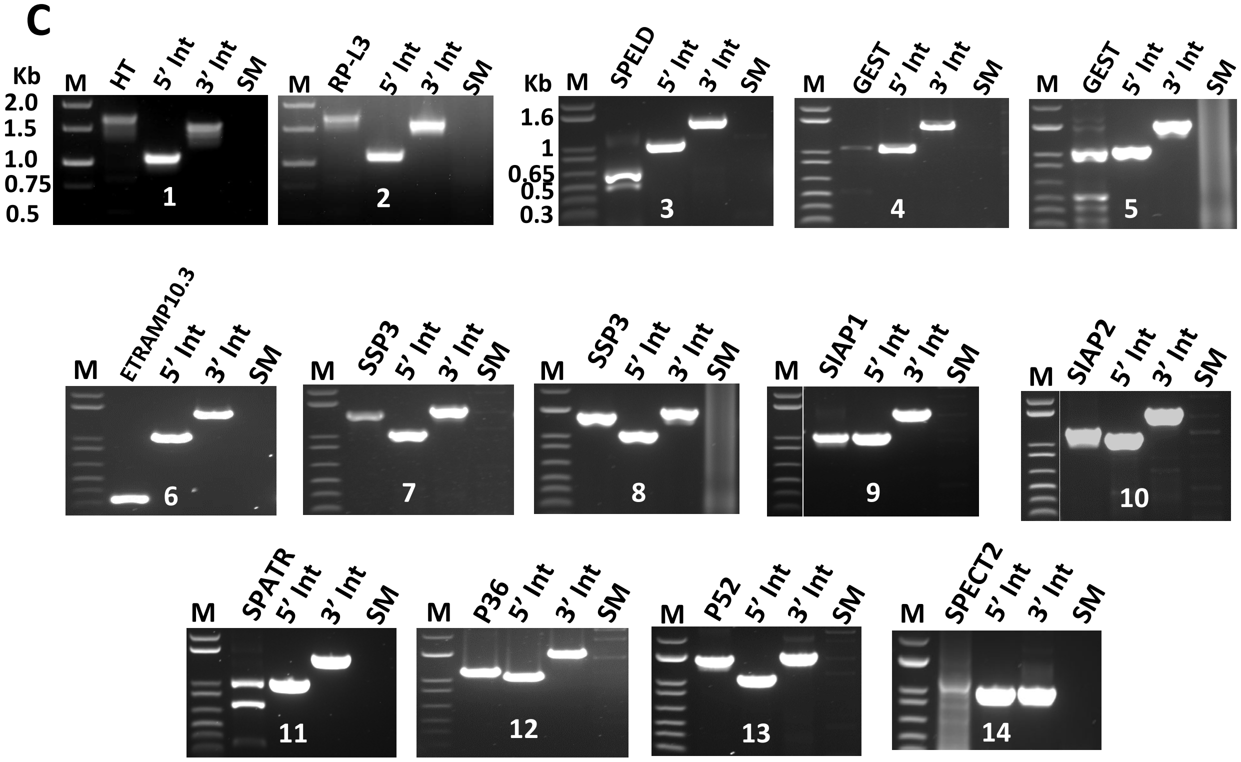

Supplement: S1 Fig — A. Schematic representation of the introduction of the P. falciparum gene expression-cassettes by double cross-over integration into the 230p locus of the P. berghei ANKA GIMO-230p mother line by GIMO-transfection. The P. falciparum gene is under control of the P. berghei uis4 regulatory sequences (5’UTR and 3’UTR). The construct also contains a gfp::luc fusion reporter gene under the constitutive Pbeef1a promoter. Black arrows: location of PCR primers used for diagnostic PCR-analysis (panel C). The table shows the 14 chimeric parasite lines generated (see S1 Table for details). orf, open reading frame; int, integration. B. Genotype analysis of the 14 chimeric parasite lines parasites by Southern analysis of chromosomes (chr.) separated by pulsed-field gel electrophoresis to confirm integration of the DNA constructs in the GIMO locus (230p on chr. 3), shown as the absence of the hdhfr::yfcu selectable marker (SM) cassette in cloned chimeric parasites by hybridisation of chr. with the hdhfr probe. Chromosomes are also hybridized to a control probe recognising chr. 5. As an additional control (ctrl), parasite line 2117cl1 is used with the hdhfr::yfcu SM integrated into chr. 3. C. Diagnostic PCR analysis of the 14 chimeric parasite lines confirming correct integration of the P. falciparum antigen expression cassettes. Correct integration in all lines is shown by the presence of the P. falciparum gene coding sequence, absence of the hdhfr::yfcu SM, and the correct integration of the construct into the genome at both the 5’and 3’regions (5’ int and 3’ int). See panel A for the location of the primers. Primer details, sequences and the expected PCR product sizes are shown in S3 Table. (PDF) [file pone.0254498.s001.pdf]
