# Supplementary figures and images for: Screening of viral-vectored P. falciparum pre-erythrocytic candidate vaccine antigens using chimeric rodent parasites
Source: PLoS One. 2021 Jul 12;16(7):e0254498. doi: 10.1371/journal.pone.0254498 (PMC8274855; doi:10.1371/journal.pone.0254498)

A

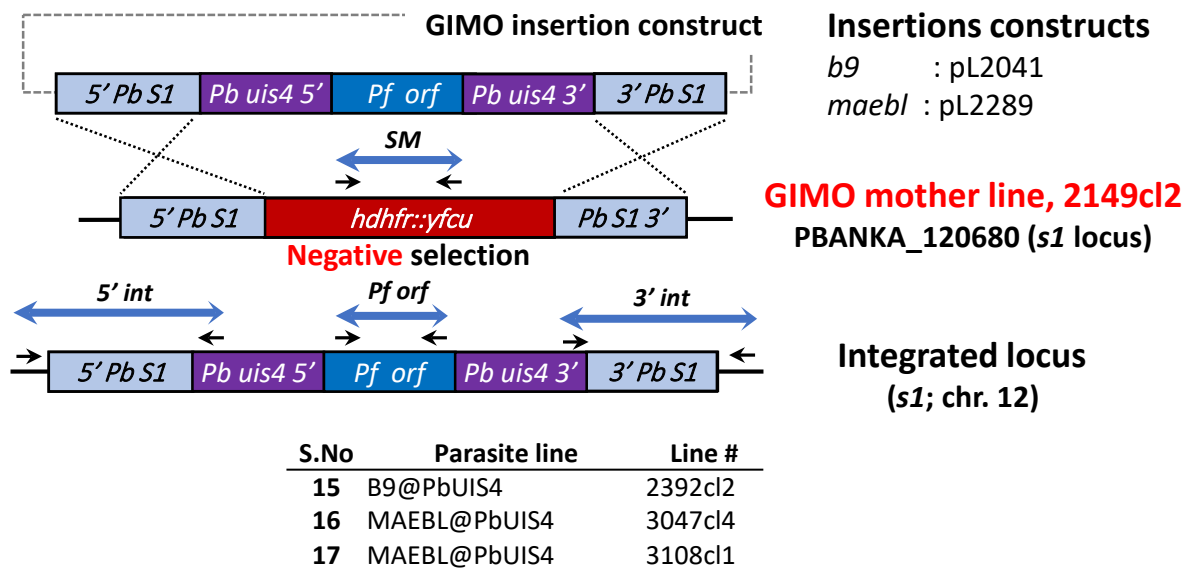

B

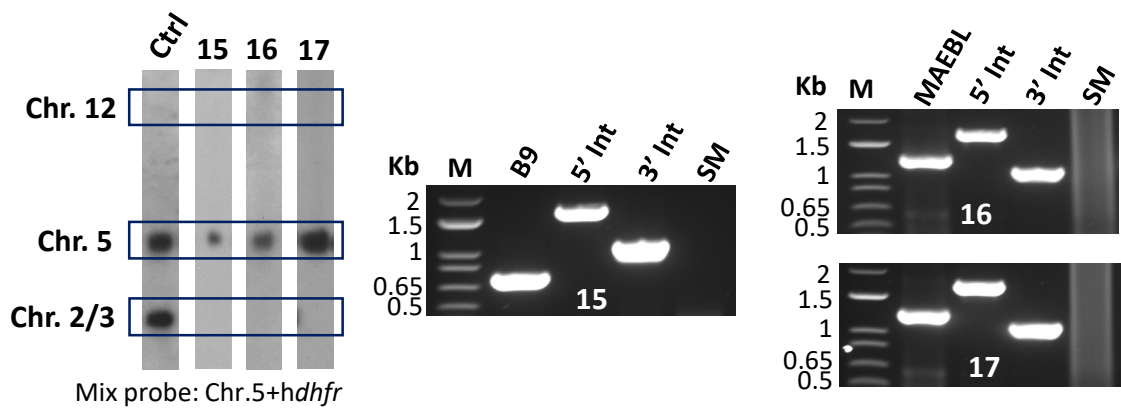

Supplement: S2 Fig — A. Schematic representation of the introduction of the P. falciparum expression-cassette (b9/maebl) by double cross-over integration into the s1 locus of P. berghei ANKA GIMO-s1 mother line by GIMO-transfection. The P. falciparum gene is under control of the P. berghei uis4 regulatory sequences (5’UTR and 3’UTR). Black arrows: location of PCR primers used for diagnostic PCR-analysis (panel B). The table shows different chimeric parasites generated. Orf, open reading frame; int, integration. B. Left: Genotype analysis of chimeric parasite lines by Southern analysis of chromosomes (chr.) separated by pulsed-field gel electrophoresis (PFGE). The correct integration of construct in the chimeric lines was confirmed by showing the absence of the hdhfr::yfcu selectable marker (SM) cassette in cloned chimeric parasites by hybridisation of chr. with the hdhfr probe. Chromosomes are also hybridized to a control probe recognising chr. 5. As an additional control (ctrl), parasite line 2117cl1 is used with the hdhfr::yfcu SM integrated into chr. 3. Right: Diagnostic PCR analysis confirms the correct integration of the P. falciparum b9 and maebl expression cassettes in the chimeric parasites. Correct integration is shown by the presence of the Pfb9 or Pfmaebl orf, absence of the hdhfr::yfcu SM, and the correct integration of the construct into the genome at both the 5’ and 3’ regions (5’int and 3’int). See panel A for the location of the primers. Primer details, sequences and the expected PCR product sizes are shown in S3 Table. (PDF) [file pone.0254498.s002.pdf]

A

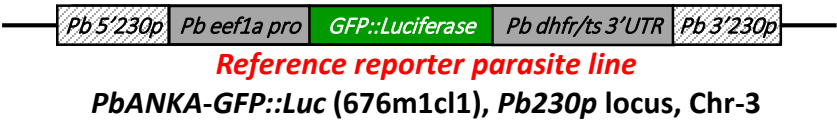

B

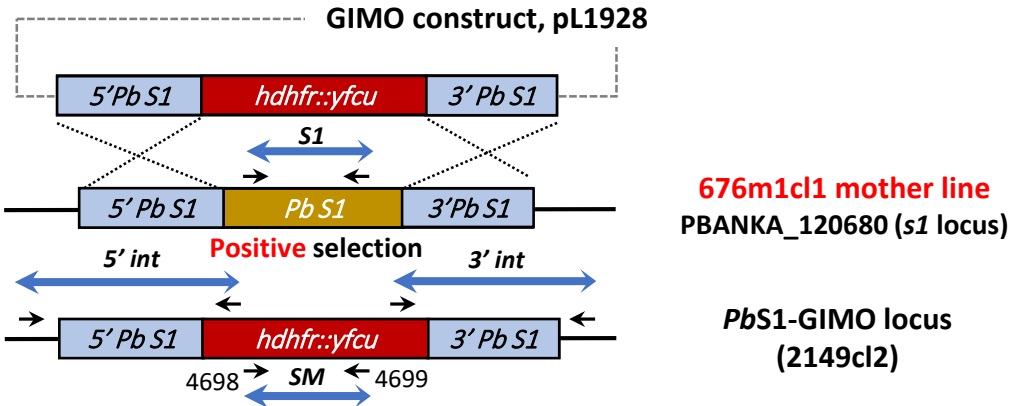

C

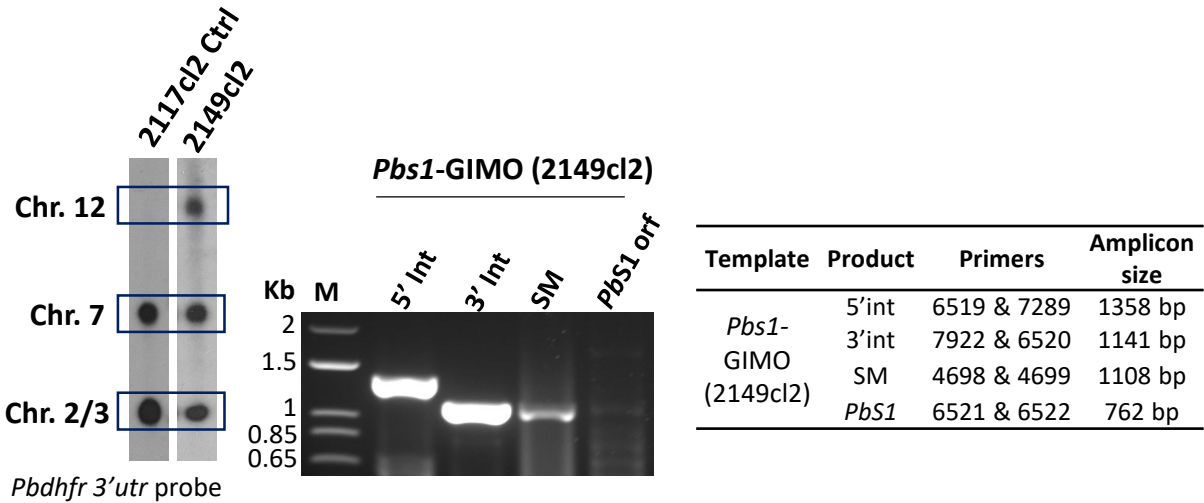

Supplement: S4 Fig — A. Schematic representation of the Pb230p locus of the reference reporter P. berghei ANKA parasite 676cl1 which was used to generate the chimeric Pb-Pfspect2(r) parasite line (see B). This parental line contains a gfp-luciferase fusion reporter gene under the constitutive Pbeef1a promoter and is selectable marker (SM) free. The reporter-cassette is integrated into the neutral 230p locus in chromosome 3. B. Schematic representation of the generation of the Pbs1 GIMO. The GIMO deletion-construct (pL1928) was used to replace the Pbs1 coding sequence (CDS) with the positive/negative selectable marker (SM; hdhfr::yfcu) cassette, resulting in the generation of the Pbs1 GIMO (line 2149cl2) after positive selection with pyrimethamine. The construct pL1928 targets the Pbs1 gene by double cross-over homologous recombination. After genotyping and confirmation of correct construct integration, this line was cloned by limiting dilution. C. Left: Genotype analysis of Pbs1 GIMO parasites by Southern analysis of chromosomes (chr.) separated by pulsed-field gel electrophoresis (PFGE) (left) and diagnostic PCR analysis (right). Hybridisation of PFG-separated chr. of Pbs1 GIMO with a 3’ UTR Pbdhfr/ts probe. This probe recognizes the construct integrated into chr. 12, the endogenous Pbdhfr/ts gene at chromosome 7 and the gfp-luciferase reporter cassette at chr. 3. As an additional control (ctrl), parasite line 2117cl1 is used with a construct containing the 3’ UTR Pbdhfr/ts integrated into chr. 3. Diagnostic PCR analysis confirms the deletion of Pbs1 in Pbs1 GIMO. Correct integration is shown by the absence of Pbs1 orf, the presence of hdhfr::yfcu SM and the correct integration of the construct into the genome at both the 5’ and 3’ regions (5’int and 3’int; see B for primer numbers and locations). Expected PCR product sizes and the primer numbers are listed in the Table in the Figure and primers sequences are shown in S3 Table. (PDF) [file pone.0254498.s004.pdf]
