# Supplementary material for: Screening of viral-vectored P. falciparum pre-erythrocytic candidate vaccine antigens using chimeric rodent parasites
Source: PLoS One. 2021 Jul 12;16(7):e0254498. doi: 10.1371/journal.pone.0254498 (PMC8274855; doi:10.1371/journal.pone.0254498)
Supplement: S3 Fig — A. Schematic representation of the Pb230p locus of the reference reporter P. berghei ANKA parasite 676cl1 which was used to generate the chimeric Pb-Pfspect2(r) parasite line (see B). This parental line contains a gfp-luciferase fusion reporter gene under the constitutive Pbeef1a promoter and is selectable marker (SM) free. The reporter-cassette is integrated into the neutral 230p locus in chromosome 3. B. Schematic representation of the generation of the chimeric line Pb-Pfspect2(r) (line 3162cl2). First step: the GIMO deletion-construct (construct 1; pL2318) was used to replace the Pbspect2 open reading frame (orf) with the positive/negative selectable marker (SM; hdhfr::yfcu) cassette, resulting in the generation of the PbΔspect2 (line 3144cl3) after positive selection with pyrimethamine. Construct 1 targets the Pbspect2 gene by double cross-over homologous recombination. After genotyping and confirmation of correct construct integration, this line was cloned by limiting dilution. Int, integration. Second step: The GIMO insertion construct (construct 2; pL2319) was used to replace the SM in the PbΔspect2 GIMO line with the Pfspect2 orf, resulting the generation of line Pb-Pfspect2(r) (line 3162cl2) after negative (5-FC) selection. Construct 2 integrates by double cross-over homologous recombination using the same targeting regions employed in construct 1, resulting in the Introduction of the Pfspect2 orf under the control of Pbspect2 regulatory sequences. Black arrows: number and location of primers used for diagnostic PCR (panel C). C. Left: Genotype analysis of PbΔspect2 and Pb-Pfspect2(r) parasites by Southern analysis of chromosomes (chr.) separated by pulsed-field gel electrophoresis (PFGE). Hybridisation of PFG-separated chr. of PbΔspect2 with a mixture of hdhfr and a probe specific for chr. 5 confirms integration of construct 1 into the Pbspect2 gene on chr. 10 in Pbspect2-GIMO (line 18). The correct integration of construct 2 in Pb-Pfspect2(r) (line 19) w [file pone.0254498.s003.pdf]

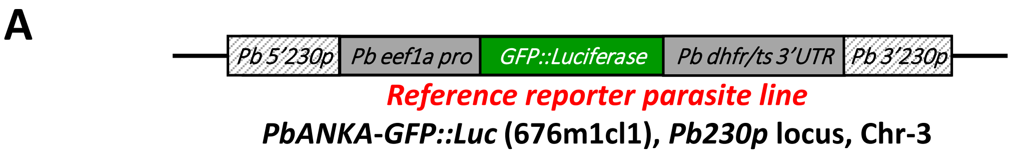

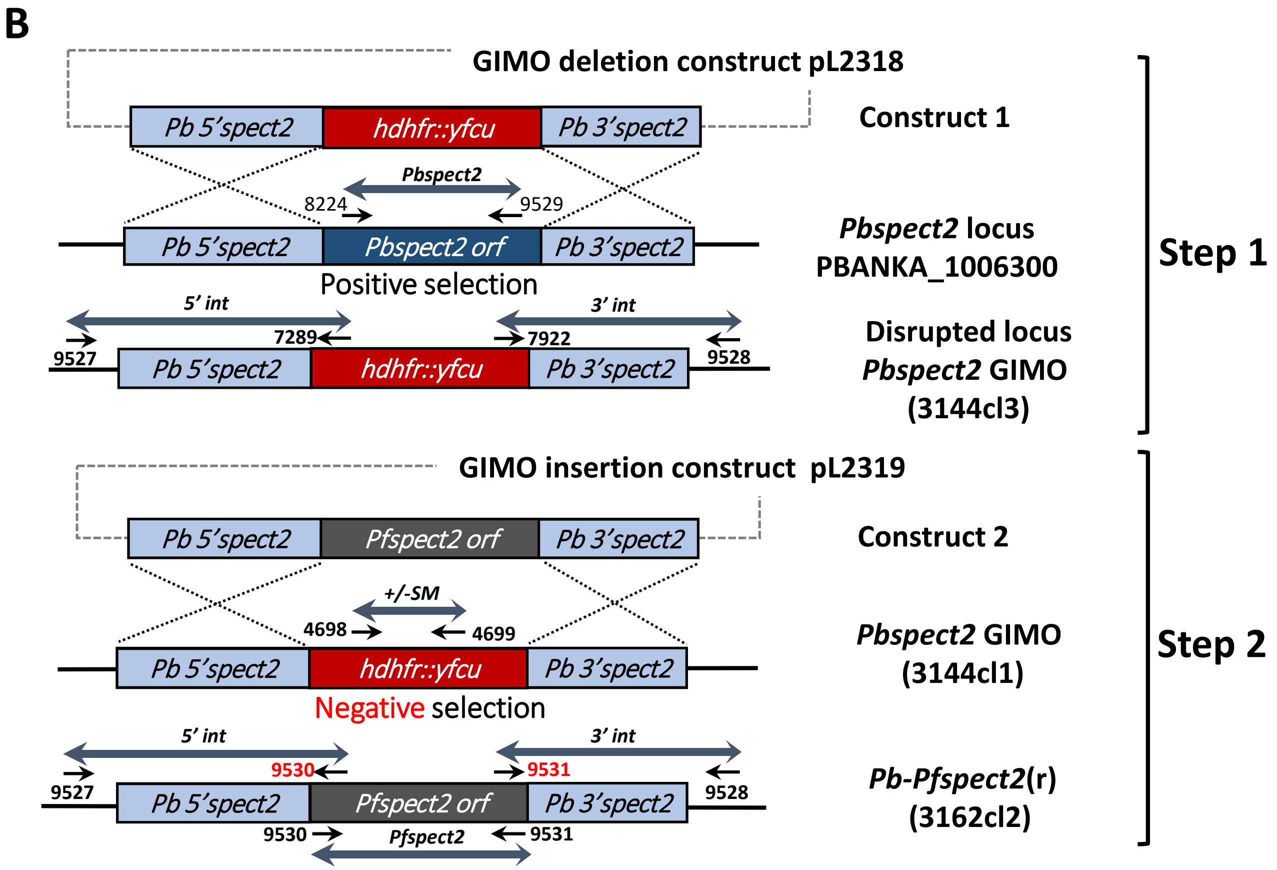

S.No	Parasite line	Line #
**18**	*Pbspect2*-GIMO	3144cl3
**19**	*Pb-Pf*SPECT2 (r)	3162cl2

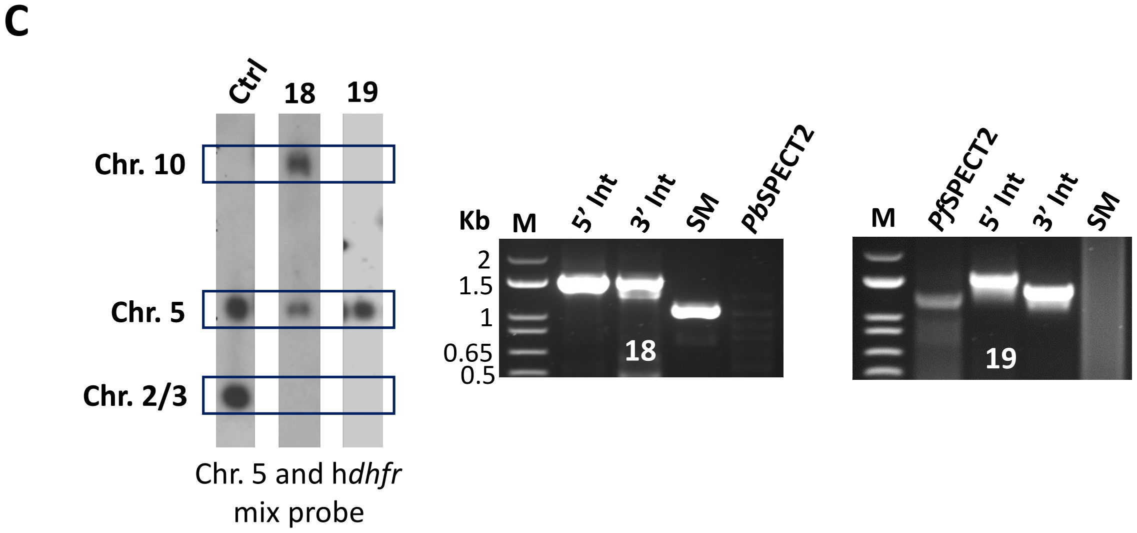

D

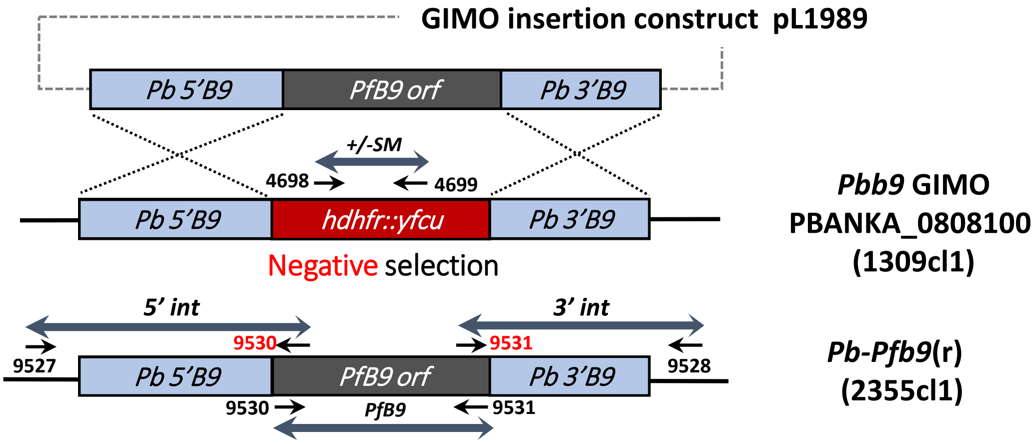

S.No	Parasite line	Line #
**20**	*Pb-Pf*B9 (r)	2355cl1

E

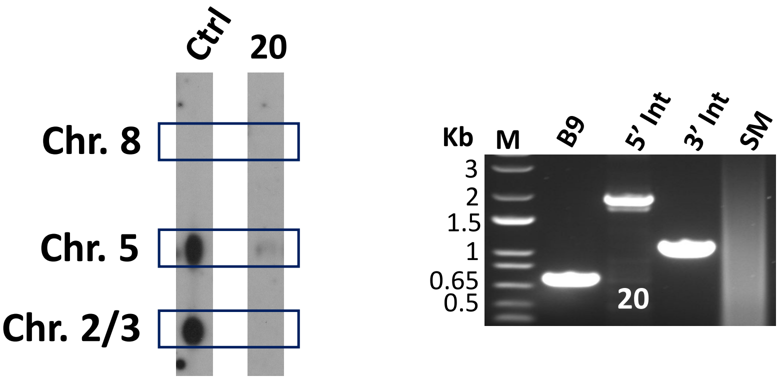

Chr. 5 and h*dhfr* mix probe
