# Supplementary material for: Screening of viral-vectored P. falciparum pre-erythrocytic candidate vaccine antigens using chimeric rodent parasites
Source: PLoS One. 2021 Jul 12;16(7):e0254498. doi: 10.1371/journal.pone.0254498 (PMC8274855; doi:10.1371/journal.pone.0254498)
Supplement: S5 Fig — A. names and line numbers of 20 transgenic parasite lines described in this study (see S1 Table for full details of the lines). B. Full length agarose gel images containing PCR fragments of the different chimeric P. berghei lines. (see S1–S4 Figs for description of the primers used for the different fragments). C. Autoradiographs of Southern analysis of chromosomes (chr.) separated by pulsed-field gel electrophoresis (PFGE). Red boxes indicate the different chimeric lines. See Table in panel A for the numbers of the chimeric lines. Ctrl: control line. (PDF) [file pone.0254498.s005.pdf]

A

S.No	Chimeric parasite	Line #	S.No	Chimeric parasite name	Line #
**1**	HT@PbUIS4	2409cl4	**12**	P36@PbUIS4	3029cl1
**2**	RP-L3@PbUIS4	2411cl1	**13**	P52@PbUIS4	3032cl1
**3**	SPELD@PbUIS4	2887cl1	**14**	SPECT2@PbUIS4	3039cl1
**4**	GEST@PbUIS4	2888cl4	**15**	B9@PbUIS4	2392cl2
**5**	GEST@PbUIS4	3015cl1	**16**	MAEBL@PbUIS4	3047cl4
**6**	ETRAMP@PbUIS4	2891cl1	**17**	MAEBL@PbUIS4	3108cl1
**7**	SSP3@PbUIS4	2895cl1	**18**	*Pb*SPECT2-GIMO	3144cl3
**8**	SSP3@PbUIS4	3018cl1	**19**	*Pb-Pf*SPECT2 (r)	3162cl2
**9**	SIAP1@PbUIS4	2909cl4	**20**	*Pb-Pf*B9 (r)	2355cl1
**10**	SIAP2@PbUIS4	2911cl1	**21**	*Pb*S1-GIMO	2149cl2
**11**	SPATR@PbUIS4	3026cl1			

B

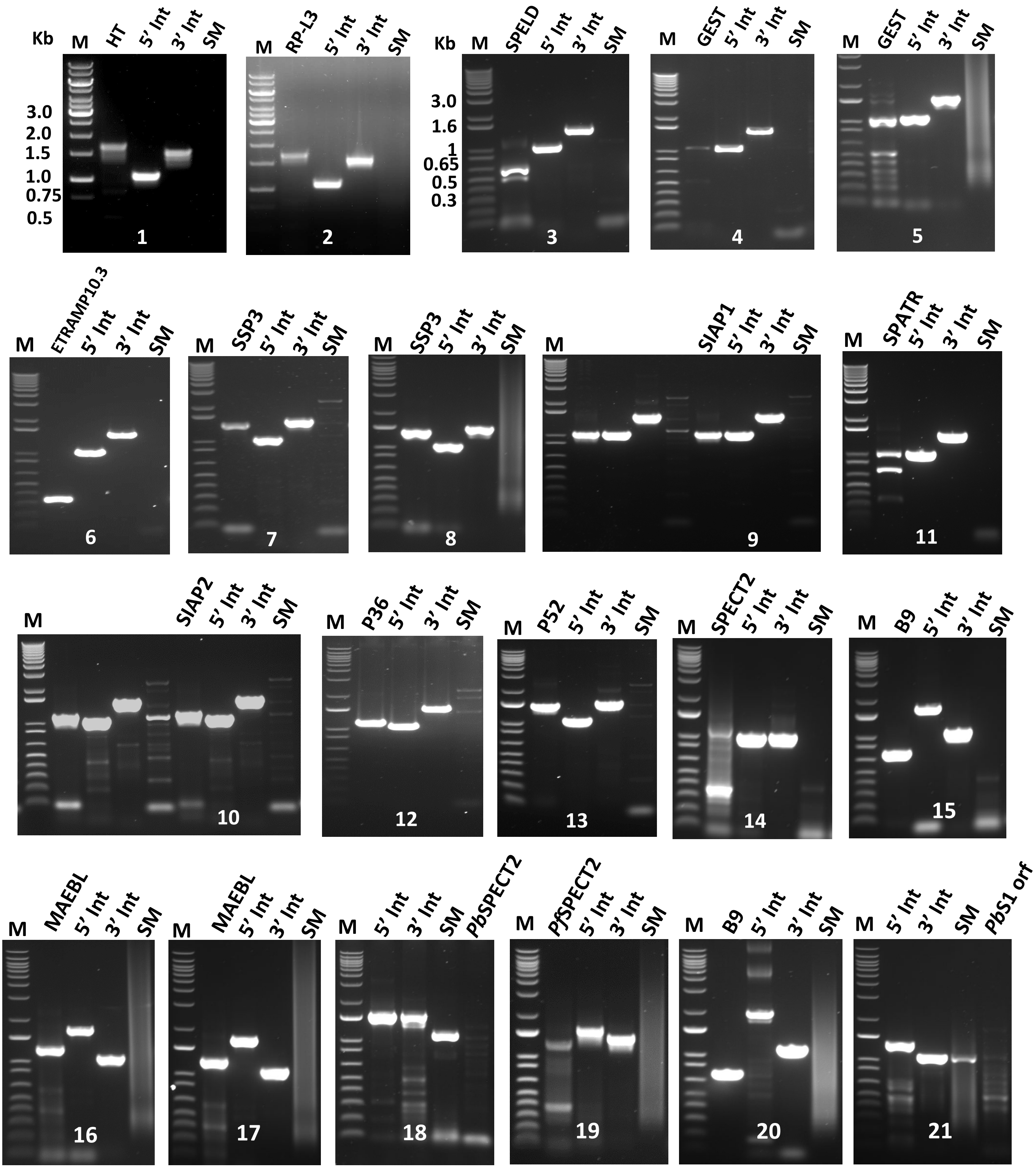

C

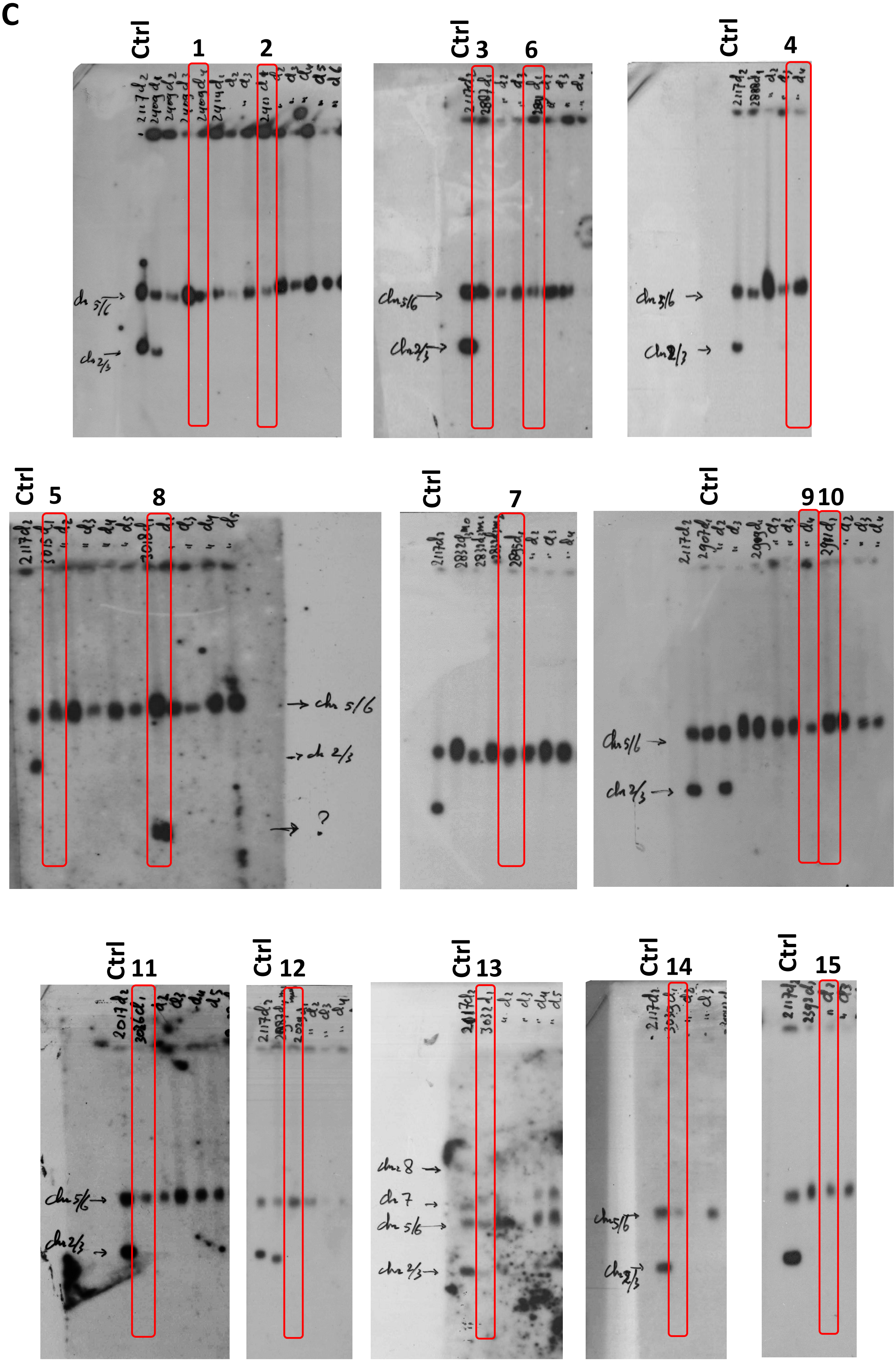
Ctrl
1
2
Ctrl
3
6
Ctrl
4
Ctrl
5
8
Ctrl
7
Ctrl
9
10
Ctrl
11
Ctrl
12
Ctrl
13
Ctrl
14
Ctrl
15

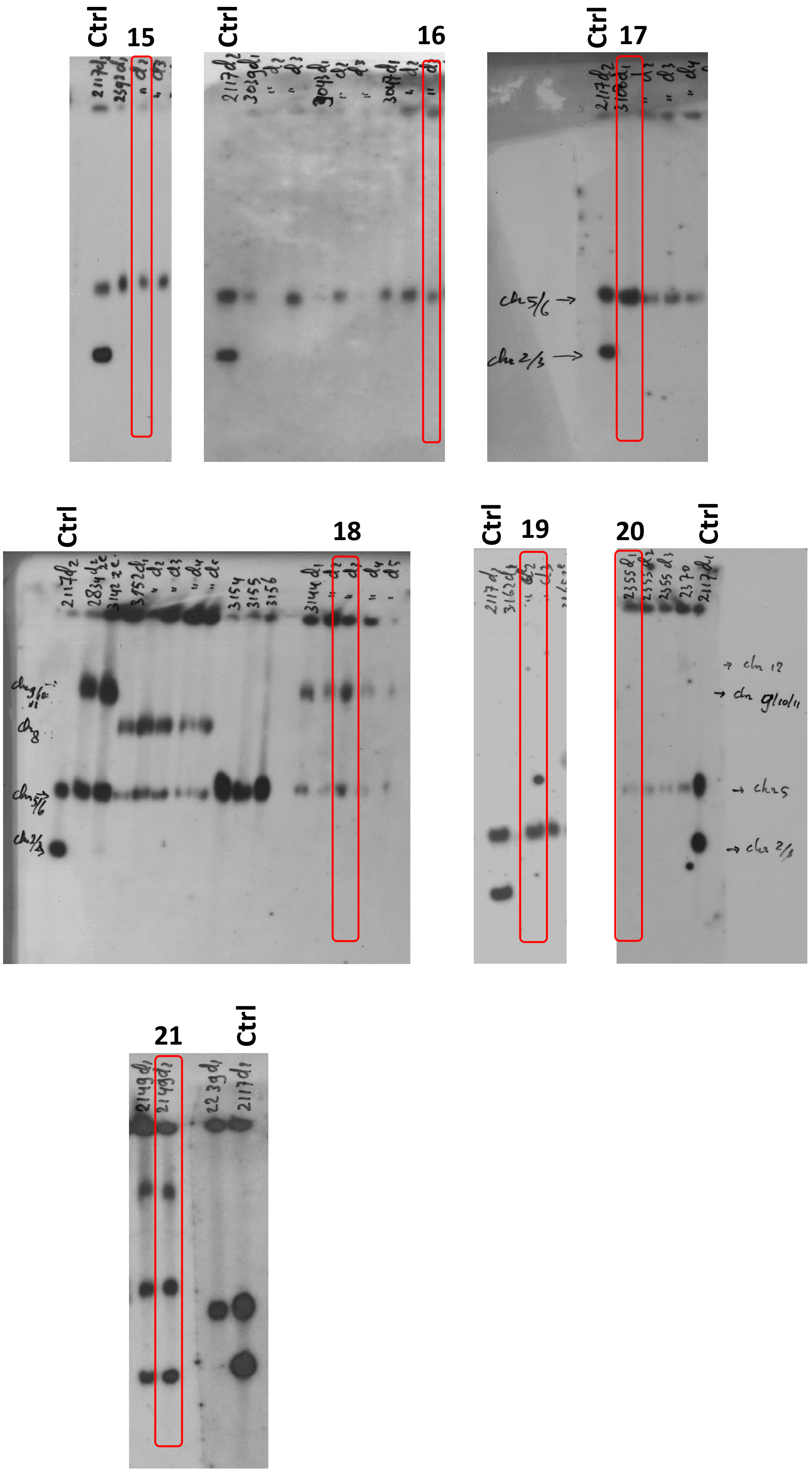

Ctrl
15
Ctrl
16
Ctrl
17
Ctrl
18
Ctrl
19
20
Ctrl
21
Ctrl
