# Supplementary material for: Screening of viral-vectored P. falciparum pre-erythrocytic candidate vaccine antigens using chimeric rodent parasites
Source: PLoS One. 2021 Jul 12;16(7):e0254498. doi: 10.1371/journal.pone.0254498 (PMC8274855; doi:10.1371/journal.pone.0254498)
Supplement: S3 Table — (DOCX) [file pone.0254498.s008.docx]

**S3 Table: List of primers**

**Primers used to generate constructs**

| **DNA Construct** | **Primer code** | **Sequence** | **Enzymes** | **Product (bp)** | **Primer description** |
| --- | --- | --- | --- | --- | --- |
| pL2005 | 7169 | TATCCTGCAGGGTGATAGTGTAGATTTTTTTGTTTGAC | PstI | 1493 | *Pb*UIS4 promoter forward |
|  | 7170 | ATAAGAATGCGGCCGCAGACGTAATAATTATGTGCTGAAAGG | NotI |  | *Pb*UIS4 promoter reverse |
|  | 7171 | CGGATATCTATAATTCATTATGAGTAGTGTAATTCAG | EcoRV | 1008 | *Pb*UIS4 *3’utr* forward |
|  | 7173 | GGCCGGTACCTTTCGCTTTAATGCTTGTCATC | KpnI |  | *Pb*UIS4 *3’utr* reverse |
| pL2281 | 7291 | CCGGGGTACCCCCAGCTTAATTCTTTTCGAGCTC | KpnI | 3460 | GFP-Luc cassette forward |
|  | 7292 | CCGGGGTACCTTGAAGGAAAAAACATCATTTGTG | KpnI |  | GFP-Luc cassette reverse |
| pL2041 | 7225 | CGGGATCCGAGGGATGAATAGGGGATGGC | BamHI | 3239 | *Pfb9* full length forward |
|  | 7226 | GTGTCACCGGCGCACTTCAACATTACTAGAAGAATGTGATAC | SgrAI |  | *Pfb9* full length reverse |
| pL2050 | 1063 | ATAAGAATGCGGCCGCATGACCAAGAGCAGCAAGGAC | NotI | 1540 | *Pfht* full length forward |
|  | 1064 | GGGGATATCTCACACCACGGACTTGGTCATGTG | EcoRV |  | *Pfht* full length reverse |
| pL2051 | 1065 | ATAAGAATGCGGCCGCATGAGCCACCGGAAGTTCG | NotI | 1161 | *Pfrp-l3* full length forward |
|  | 1066 | GGGGATATCTTAGGCCTTCAGGTCCTTCTTCAG | EcoRV |  | *Pfrp-l3* full length reverse |
| pL2225 | 8538 | GATGCGGCCGCATGTGCAGCACATATTCCGTTG | NotI | 626 | *Pfspeld* full length forward |
|  | 8539 | CAGGATATCCATCTTATGGTACAAGGACGG | BsaBI |  | *Pfspeld* full length reverse |
| pL2226 | 8605 | GATGCGGCCGCATGAATAAATTTACATATTGTATTAC | NotI | 1051 | *Pfgest* full length forward |
|  | 8606 | GACGATATCCATCTTATTGTAATAGGGCTTTTTCTTG | BsaBI |  | *Pfgest* full length reverse |
| pL2227 | 8603 | GTAGCGGCCGCATGAAGGTTTCTAGGCATACCGTTC | NotI | 327 | *Pfetramp10.3* full length forward |
|  | 8604 | GACGATATCCATCTTATTCATCTTTGTCTTTGTCC | BsaBI |  | *Pfetramp10.3* full length forward |
| pL2229 | 8613 | GATGCGGCCGCTGGTTAAGATGCCGAACATGTTG | NotI | 1386 | *Pfssp3* full length forward |
|  | 8614 | GAAGATATCCATCCTATTTTTTACTCAAATTTGAATCG | BsaBI |  | *Pfssp3* full length reverse |
| pL2232 | 8607 | GATGCGGCCGCATGGAAGGCTTTGTTGCTTTGCTG | NotI | 2955 | *Pfsiap1* full length forward |
|  | 8608 | GGACCCGGGTTAATCATTTTGAACGTAAACAC | SmaI |  | *Pfsiap1* full length reverse |
| pL2233 | 8609 | GATGCGGCCGCATGAACATGTACGTAATCTATTACTAC | NotI | 1167 | *Pfsiap2* full length forward |
|  | 8610 | GAGGATATCCATCTTATTGTATTTTTTTAGATATCATAGC | BsaBI |  | *Pfsiap2* full length reverse |
| pL2228 | 8611 | GATGCGGCCGCATGAAAAAAAGTCGTTTTTTGC | NotI | 1069 | *Pfspatr* full length forward |
|  | 8612 | GACGATATCCATCTTAACATGAACGCGTTTGTTCC | BsaBI |  | *Pfspatr* full length reverse |
| pL2282 | 8868 | GATGCGGCCGCATGGCTTATAATATTTGGG | NotI | 1140 | *Pfp36* full length forward |
|  | 8869 | GACGATATCCATCCTCATCTAACTTTCTACAG | BsaBI |  | *Pfp36* full length reverse |
| pL2283 | 8870 | GATGCGGCCGCATGTATGTATTGGTGCTTATTC | NotI | 1347 | *Pfp52* full length forward |
|  | 8871 | GACGATATCTATTAATATATTAAGTTATTATGTTG | EcoRV |  | *Pfp52* full length reverse |
| pL2286 | 1293 | ATAAGAATGCGGCCGCATGAAGCTGAGAATCCTGAAGAAAC | NotI | 2532 | *Pfspect2/plp1* fulll length forward |
|  | 1294 | ATCCCGGGTCATCACTTGTTGTTTTTGCACAGCATGTAG | XmaI |  | *Pfspect2/plp1* fulll length reverse |
| pL2319 | 1289 | ATCCGCGGATGAAGCTGAGAATCCTGAAGAAAC | SacII | 2532 | *Pfspect2/plp1* fulll length forward |
|  | 1290 | CCGGGGTACCTCATCACTTGTTGTTTTTGCACAGCATGTAG | KpnI |  | *Pfspect2/plp1* fulll length reverse |
| pL2289 | 8540 | TCTGCGGCCGCATGGGGGTATTGAAACAT | NotI | 6762 | *Pfmaebl* full length forward |
|  | 8543 | GACGATATCCATCTCAAACCTATAAATTTAA | BsaBI |  | *Pfmaebl* full length reverse |
| pL2318 | 1285 | CGCAAGCTTTGTGTAAATTGAAAGAAAGATTGAAATGGAAG | HindIII | 1291 | *Pbspect2/plp1* HR1 forward |
|  | 1286 | ATCCGCGGTCAATTACATATATTTGTAAATATATACATTTTCCTTTTC | SacII |  | *Pbspect2/plp1* HR1 reverse |
|  | 1287 | CCGGGGTACCAACACAAATCAATTTACACCTCTTCATG | KpnI | 985 | *Pbspect2/plp1* HR2 forward |
|  | 1288 | ATAAGAATGCGGCCGCTATAGAAAAGATGTATGTATAATACCGAAAATTGG | NotI |  | *Pbspect2/plp1* HR2 reverse |
| pL1989 | 7225 | CGGGATCCGAGGGATGAATAGGGGATGGC | BamHI | 3201 | *Pfb9* full length forward |
|  | 7226 | GTGTCACCGGCGCACTTCAACATTACTAGAAGAATGTGATAC | SgrAI |  | *Pfb9* full length reverse |
| pL1928 | 1003 | CATGGGCCCACCATGCTTTGTCTGAGAGTG | *ApaI* | 766 | *Pbs1* 5' target region forward |
|  | 1004 | AAGGCCTGGTACCATACTGTTCTTCCCAATGGATC | *StuI /KpnI* |  | *Pbs1* 5' target region reverse |
|  | 1005 | ATAAGAATGCGGCCGCCTGCAGCATTCAAATGCTTGAAGGCGATG | *Ava1/NotI/PstI* | 820 | *Pbs1* 3' target region forward |
|  | 1006 | ACATGGCGCCAAGCTTATGGCACATGGATCGAACAG | *KasI/HindIII* |  | *Pbs1* 3' target region reverse |

**Primers used for integration PCRs**

| **Line Number** | **Primer code** | **Sequence** | **Product size (bp)** | **Primer description** | **Remarks** |
| --- | --- | --- | --- | --- | --- |
| All lines | 4698 | GTTCGCTAAACTGCATCGTC | 1108 | SM forward | Selectable marker |
|  | 4699 | GTTTGAGGTAGCAAGTAGACG |  | SM reverse |  |
| Lines generated in the reference line - 1596cl1 | 5509 | GGTGATGGAATGGCAAAATCTG | 1079 | *Pb230p* integration forward | 5’ integration |
|  | 8811 | ACGGATGCATAGAAGCATTCCTTATTG |  | *PbUIS4* promoter reverse |  |
|  | 6833 | GAAAGGTCTTACCGGAAAACTC | 1544 | *PbUIS4* 3’utr forward | 3’ integration |
|  | 5511 | AGTGACTTTCAGTGAAATCGC |  | *Pb230p* integration reverse |  |
| 2409cl4 | VVCF22 | ATCAAGGAAACAAAGGGCGG | 1,758 | *Pfht* integration forward | *Pf orf*  *Pf orf* |
|  | 1052 | ACAAGAATTGGGACAACTCCAG |  | GFP reverse |  |
| 2411cl1 | VVCF23 | CCCTGAAGTTCATCGACACC | 1,775 | *Pfrp-l3* integration forward |  |
|  | 1052 | ACAAGAATTGGGACAACTCCAG |  | GFP reverse |  |
| 2887cl1 | 8538 | GATGCGGCCGCATGTGCAGCACATATTCCGTTG | 651 | *Pfspeld orf* forward |  |
|  | 8539 | CAGGATATCCATCTTATGGTACAAGGACGG |  | *Pfspeld orf* reverse |  |
| 2888cl4 and 3015cl1 | 8605 | GATGCGGCCGCATGAATAAATTTACATATTGTATTAC | 1075 | *Pfgest orf* forward |  |
|  | 8606 | GACGATATCCATCTTATTGTAATAGGGCTTTTTCTTG |  | *Pfgest orf* reverse |  |
| 2891cl1 | 8603 | GTAGCGGCCGCATGAAGGTTTCTAGGCATACCGTTC | 351 | *Pfetramp10.3 orf* forward |  |
|  | 8604 | GACGATATCCATCTTATTCATCTTTGTCTTTGTCC |  | *Pfetramp10.3 orf* reverse |  |
| 2895cl1 and 3018cl1 | 8613 | GATGCGGCCGCTGGTTAAGATGCCGAACATGTTG | 1418 | *Pfssp3 orf* forward |  |
|  | 8614 | GAAGATATCCATCCTATTTTTTACTCAAATTTGAATCG |  | *Pfssp3 orf* reverse |  |
| 2909cl4 | 8696 | GTCTGCTCATGATAAAAATGCTAGATCG | 1074 | *Pfsiap1 orf* forward |  |
|  | 8697 | GCATAACCATGTTTAGCACAACCTTCAGAACC |  | *Pfsiap1 orf* reverse |  |
| 2911cl1 | 8609 | GATGCGGCCGCATGAACATGTACGTAATCTATTACTAC | 1191 | *Pfsiap2 orf* forward |  |
|  | 8610 | GAGGATATCCATCTTATTGTATTTTTTTAGATATCATAGC |  | *Pfsiap2 orf* reverse |  |
| 3026cl1 | 8611 | GATGCGGCCGCATGAAAAAAAGTCGTTTTTTGC | 1069 | *Pfspatr orf* forward |  |
|  | 8612 | GACGATATCCATCTTAACATGAACGCGTTTGTTCC |  | *Pfspatr orf* reverse |  |
| 3029cl3 | 8868 | GATGCGGCCGCATGGCTTATAATATTTGGG | 1140 | *Pfp36 orf* forward |  |
|  | 8869 | GACGATATCCATCCTCATCTAACTTTCTACAG |  | *Pfp36 orf* reverse |  |
| 3032cl1 | 8870 | GATGCGGCCGCATGTATGTATTGGTGCTTATTC | 1347 | *Pfp52 orf* forward |  |
|  | 8871 | GACGATATCTATTAATATATTAAGTTATTATGTTG |  | *Pfp52 orf* reverse |  |
| 3039cl1 | 9583 | GACCTGAGCACCCTGCAGCCCGT | 1217 | *Pfspect2 orf* forward |  |
|  | 9584 | AGGATGGAGTAGTCGTTGGGGCACTTC |  | *Pfspect2 orf* reverse |  |
|  | | | | | |
| 2149cl2 | 6519 | GGTTAGCATTCAATCTGTCTG | 1358 | *Pbs1* integration forward | 5’ integration |
|  | 7289 | TAAAGCACAATATCTAGGATACTAC |  | *Pbef1a* promoter reverse |  |
|  | 7922 | GTCTCTTCAATGATTCATAAATAGTTGG | 1141 | *Pbdhfr 3’utr* forward | 3’ integration |
|  | 6520 | GTGCAAATTTCGATGGGCTC |  | *Pbs1* integration reverse |  |
|  | 6521 | AACTGCAACTATGCTCATGTG | 762 | *Pbs1* *orf* forward | Absence of *Pbs1* *orf* |
|  | 6522 | GCTACAATTCGTACTTCCAC |  | *Pbs1* *orf* reverse |  |
|  | | | | | |
| 2392cl2 | 7349 | ATATGGAAGAATCTGTGATGAGTG | 768 | *Pfb9 orf* forward | *Pfb9 orf* |
|  | 5503 | ATGGGCCCCTCATATATTAGAAAAAAATGATTCCTTG |  | *Pfb9 orf* reverse |  |
|  | 6519 | GGTTAGCATTCAATCTGTCTG | 1764 | *Pbs1* integration forward | 5’ integration |
|  | 7257 | CCACATATGGCTCATTATAGGGTG |  | *Pfb9 orf* forward |  |
|  | 8811 | ACGGATGCATAGAAGCATTCCTTATTG | 1112 | *Pfb9 orf* reverse | 3’ integration |
|  | 6520 | GTGCAAATTTCGATGGGCTC |  | *Pbs1* integration reverse |  |
|  | | | | | |
| 3047cl3 and 3108cl1 | 8692 | CACATGGTCTTGGGGGTAGATTAGGTGC | 1278 | *Pfmaebl orf* forward | *Pfmaebl orf* |
|  | 8693 | CGTCATTTCCATATTCACCTTCCTTTTGCC |  | *Pfmaebl orf* reverse |  |
|  | 6519 | GGTTAGCATTCAATCTGTCTG | 1764 | *Pbs1* integration forward | 5’ integration |
|  | 7257 | GTAGAGAGAATTATACTTCATAACG |  | *Pb UIS4* 3’utr reverse |  |
|  | 8811 | ACGGATGCATAGAAGCATTCCTTATTG | 1112 | *Pb UIS4* promoter forward | 3’ integration |
|  | 6520 | GTGCAAATTTCGATGGGCTC |  | *Pbs1* integration reverse |  |
|  | | | | | |
| 3144cl1 | 9527 | GACGATGATTACTATTATGACTATGACAATGGTTATG | 1566 | *Pbspect2* integration forward | 5’ integration |
|  | 7289 | TAAAGCACAATATCTAGGATACTAC |  | *Pbef1a* promoter reverse |  |
|  | 5697 | TTTGTGCATGCACATGCATG | 1581 | *Pbdhfr 3’utr* forward | 3’ integration |
|  | 9528 | TACCTAATGGTAGTGAAGTTAAACATATCAACC |  | *Pbspect2* integration reverse |  |
|  | 8224 | GCAGATAAACGAATGTTCTAGTGTC | 1059 | *Pbspect2* *orf* forward | Absence of *Pbspect2* *orf* |
|  | 9529 | AGTTCCATGAGGAGATGATCCATACAATC |  | *Pbspect2* *orf* reverse |  |
|  | | | | | |
| 3162cl1 | 9583 | GACCTGAGCACCCTGCAGCCCGT | 1217 | *Pfspect2* *orf* forward | *Pfspect2 orf* |
|  | 9584 | AGGATGGAGTAGTCGTTGGGGCACTTC |  | *Pfspect2* *orf* reverse |  |
|  | 9527 | GACGATGATTACTATTATGACTATGACAATGGTTATG | 1551 | *Pbspect2* integration forward | 5’ integration |
|  | 9581 | CTTGTAGATGGACCGGTTGTTCAGCC |  | *Pfspect2* *orf* reverse |  |
|  | 9582 | AGACCCCCTTCGAGAAGTGCGCCAAG | 1337 | *Pfspect2* *orf* forward | 3’ integration |
|  | 9528 | TACCTAATGGTAGTGAAGTTAAACATATCAACC |  | *Pbspect2* integration reverse |  |

orf- open reading frame, utr – untranslated region and SM - selectable marker
